# Multinary Tetrahedrite (Cu_12–*x*–*y*_M_*x*_N_*y*_Sb_4_S_13_) Nanoparticles: Tailoring Thermal and Optical Properties with Copper-Site Dopants

**DOI:** 10.1021/acs.chemmater.3c03110

**Published:** 2024-03-25

**Authors:** Jacob E. Daniel, Christian M. Jesby, Katherine E. Plass, Mary E. Anderson

**Affiliations:** † Chemistry Department, Furman University, Greenville, South Carolina 29613, United States; ‡ Chemistry Department, Franklin & Marshall College, Lancaster, Pennsylvania 17604, United States

## Abstract

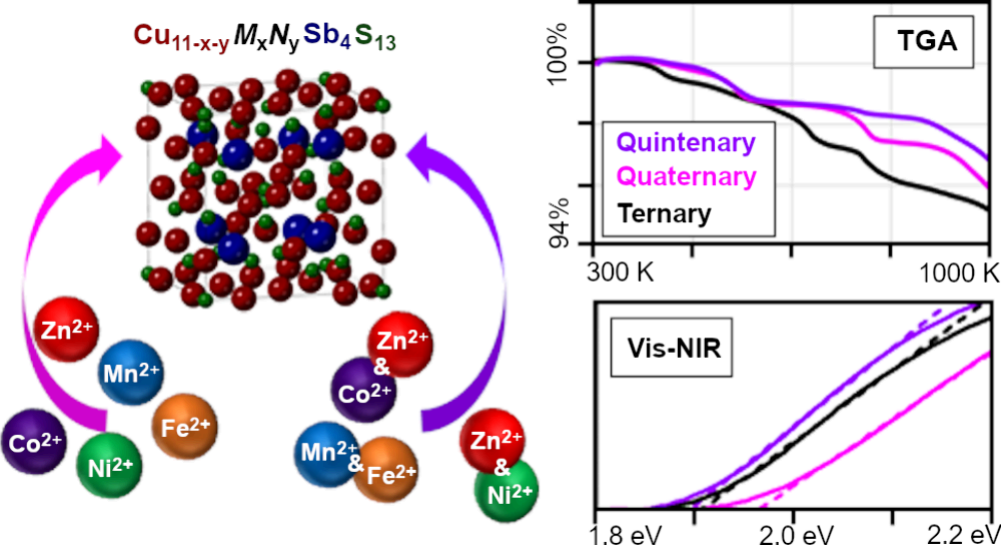

Tetrahedrite (Cu_12_Sb_4_S_13_) is an earth-abundant and nontoxic compound with prospective applications in green energy technologies such as thermoelectric waste heat recycling or photovoltaic power generation. A facile, one-pot solution-phase modified polyol method has been developed that produces high-purity nanoscale tetrahedrite products with exceptional stoichiometric and phase control. This modified polyol method is used here to produce phase-pure quaternary and quintenary tetrahedrite nanoparticles doped on the Cu-site with Zn, Fe, Ni, Mn, or Co. This is the first time that Cu-site codoped quintenary tetrahedrite and Mn-doped quaternary tetrahedrite have been produced by a solution-phase method. X-ray diffraction shows phase-pure tetrahedrite, while scanning and transmission electron microscopy show the size and morphology of the nanomaterials. Energy dispersive X-ray spectroscopy confirms nanoparticles have near-stoichiometric elemental compositions. Thermal stability of quintenary codoped tetrahedrite material is analyzed using thermogravimetric analysis, finding that codoping with Mn, Fe, Ni, and Zn increased thermal stability while codoping with cobalt decreased thermal stability. This is the first systematic study of the optical properties of quaternary and quintenary tetrahedrite nanoparticles doped on the Cu-site. Visible–NIR diffuse reflectance spectroscopy reveals that the quaternary and quintenary tetrahedrite nanoparticles have direct optical band gaps ranging from 1.88 to 2.04 eV. Data from thermal and optical characterization support that codoped tetrahedrite nanoparticles are composed of quintenary grains. This research seeks to enhance understanding of the material properties of tetrahedrite, leading to the optimization of sustainable, nontoxic, and high-performance photovoltaic and thermoelectric materials.

## 1 Introduction

Renewable energy technologies such as thermoelectric generators and photovoltaic cells are central to transitioning away from a fossil-fuel based energy economy and alleviating climate change.^1,2^ Ideal thermal and optical properties such as the thermal stability and band gap energy of a material are key for high-performance thermoelectric and photovoltaic application.^3−10^ For example, prospective materials require a high thermal stability to avoid material degradation and achieve peak performance at relevant temperatures. The metric for evaluating thermoelectric materials is the dimensionless thermoelectric figure of merit (*ZT*) given by the expression *ZT* = (*S*^2^σ/κ)*T*, where *S* is the Seebeck coefficient, σ is the electrical conductivity, κ is the thermal conductivity, and *T* is the absolute temperature.^3−7^ The efficiency of a photovoltaic material (η) is evaluated by comparing the generated power (*P*_out_) with the power of the incident photons (*P*_in_) in the equation η = *P*_*out*_/*P*_*in*_.^8−10^ In general, the ideal thermoelectric material possesses a high *S*, high σ, and low κ,^3−7^ while the ideal photovoltaic material has a band gap between 1.2 and 1.5 eV.^8−10^ Many thermoelectric and photovoltaic materials are based on narrow band gap semiconductors, namely metal chalcogenides or metal pnictides. Bi_2_Te_3_ and PbTe are applied industrially and have achieved *ZT* values of 1.6 and 2.5, respectively.^11,12^ For photovoltaics, crystalline Si (η ≈ 25%) dominates the commercial solar energy market while CdTe (η = 22.3%) and InGaAsP (η = 47.6%) thin films are emerging materials.^13−17^ A major problem with current state-of-the-art commercially and industrially applied thermoelectric and photovoltaic materials is their expensive, rare-earth, and toxic constituent elements. As a result, development and optimization of low-cost, environmentally friendly, and thermally stable materials is essential.^3,18−20^

**Figure 1:**
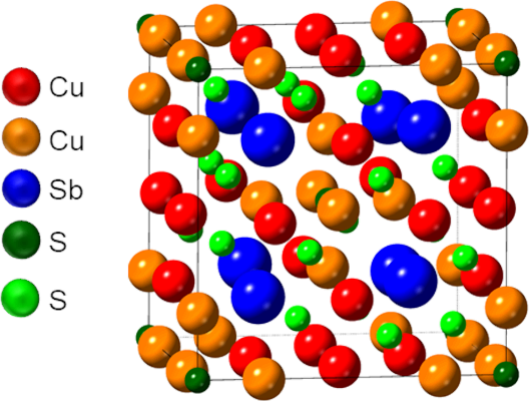
Unit cell of tetrahedrite (Cu_12_Sb_4_S_13_).^29^ The Cu tetrahedral (red) and trigonal (orange) positions relative to sulfur atoms are indicated. The Sb (blue) is trigonally coordinated to sulfur and contains a single lone pair. The S octahedral (dark green) positions are octahedrally coordinated to Cu atoms, while the S tetrahedral (light green) position is coordinated to three Cu atoms and one Sb atom.

Multinary copper sulfide materials are attractive for energy applications because of their safety, affordability, broad range of composition and stoichiometry, favorable physical properties, and varied synthetic routes.^20−26^ One such example is the ternary Cu–Sb–S family containing the p-type semiconductors chalcostibite (CuSbS_2_), skinnerite (Cu_3_SbS_3_), famatinite (Cu_3_SbS_4_), and tetrahedrite (Cu_12_Sb_4_S_13_).^20−23^ Of the four compounds, tetrahedrite is most widely studied for potential thermoelectric and photovoltaic applications because of promising physical and electronic properties.^22−28^ Tetrahedrite is a naturally occurring sulfosalt with a complex, 58-atom, cubic unit cell shown in Figure 1.^23−29^ The unit cell contains vacancy sites allowing for a range of compositions (Cu_12–14.5_Sb_4–4.5_S_13_).^24^ The complexity of the structure greatly increases phonon scattering and results in favorable thermoelectric properties, such as a low thermal conductivity of ∼0.5 W m^–1^ K^–1^ and a high electrical conductivity.^24−28^ Tetrahedrite is also a strong light absorber, with an absorption coefficient on the scale of 10^4^ cm^–1^ across the visible and the near-infrared regions. Tetrahedrite possesses a direct optical bandgap that has been predicted computationally and shown experimentally to range from 1.5 to 2.0 eV.^23,26,27^

Dopant incorporation and nanostructuring are two common strategies utilized to improve key material properties such as electronic, optical, and thermal properties^30−45^ and have been applied to improve tetrahedrite for thermoelectric and photovoltaic applications.^32−43^ Doping modifies the electronic structure, transport properties, and optical band gap of materials by modifying the density of states around the Fermi level, while also decreasing the thermal conductivity by increasing phonon scattering.^4,5,30−32^ Bulk Zn-doped and Mn-doped tetrahedrite displayed *ZT* values of 1.0 and 1.13, respectively, an increase relative to the undoped material (0.65).^33,34^ However, only a few studies in the literature have performed thermal analysis to determine high temperature stability of doped tetrahedrites alongside thermoelectric characterization.^35−37,41^ Regarding the optical properties of tetrahedrite, only one study has evaluated the effect of Cu-site doping. It found that the addition of Zn dopant shifted the band gap of the tetrahedrite to higher energies and that the magnitude of the shift depended on the amount of dopant.^38^ Nanostructuring introduces additional grain boundaries within a material but does not directly change the band gap outside of the quantum confinement regime. Nanostructuring does impact phonon scattering and photon absorption, which decreases thermal conductivity and improves photovoltaic efficiency, respectively.^4−7,39−45^ A recent publication produced nanostructured, undoped tetrahedrite with a *ZT* of 1.15, which was due to a decreased thermal conductivity and increased Seebeck coefficient.^42^ For photovoltaics, rapid charge collection is enabled by nanostructuring, which increases photon absorption and reduces recombination, a main driver of efficiency loss.^43,44^ While this has not been shown for tetrahedrite, research shows that a photovoltaic cell based on nanoscale ZnO showcased increased photovoltaic efficiency relative to a bulk counterpart.^45^ Nanostructuring undoped tetrahedrite lowers the electrical conductivity and decreases thermal stability of the material.^39−41^ Such decreases to electrical conductivity due to nanostructuring are offset by decreases to thermal conductivity.^6,7,39,40^ Furthermore, the inclusion of transition metal dopants has the potential to combat the loss in thermal stability due to nanostructuring.^35,36,41^

This study utilizes a rapid and energy efficient solution-phase modified polyol synthesis to produce ligand-free tetrahedrite with thermoelectric properties equal to or better than tetrahedrite fabricated by solid-state methods.^39−41^ Previously, the modified polyol method demonstrated tailorable stoichiometric incorporation of first-row transition metal dopants on the Cu-site to produce phase-pure quaternary tetrahedrite.^41^ Other solution-phase synthetic methods such as hot-injection^23,46−48^ and solvothermal^49−51^ procedures fabricate tetrahedrite nanoparticles coated with ligands or surfactants, which negatively impacts thermoelectric properties.^39,52^ Optical and thermoelectric properties of these ligand-coated particles have been explored;^23,46−51^ however, neither thermal stability studies nor dopant incorporation have been undertaken for these materials. Solid-state methods, such as melting and recrystallization^32−36^ or mechanical alloying,^37,53^ are energy-intensive and time-consuming methods that produce bulk tetrahedrite. These materials typically undergo thermoelectric characterization and less commonly thermal stability studies. Some research has investigated how thermoelectric properties of the bulk material are impacted by doping on the Cu-site with transition metals such as Zn, Fe, Ni, Mn, and Co.^32−37^ To better understand the impact of synthetic methods, nanostructuring, and dopant incorporation, the optical and thermal properties of tetrahedrite materials made herein by the polyol process are compared to those produced by other methods.

Thermoelectric and photovoltaic technologies often operate in high-temperature environments, making the thermal stabilities of constituent materials highly important. While nanostructuring decreases thermal conductivity and increases thermoelectric performance relative to bulk material,^4−7,39−41^ nanostructured tetrahedrite has lower thermal stability than bulk tetrahedrite.^41^ Fasana et al. found that polyol-synthesized undoped tetrahedrite nanoparticles have a significant endothermic transition at temperatures ∼100 K lower than bulk tetrahedrite. However, the doped tetrahedrite nanoparticles underwent an endothermic transition at temperatures ∼50 K higher than undoped nanoparticles, displaying improved thermal stability.^41^ Therefore, the synthesis of quintenary, codoped tetrahedrite nanoparticles is pursued as a method to further increase the thermal stability of tetrahedrite material. Codoping involves introducing two coexisting dopants into the tetrahedrite crystal lattice, but studies thus far only evaluated the impact of codoping on thermoelectric properties.^54−58^ A quintenary tetrahedrite with Zn and Ni as Cu-site codopants displayed improved thermoelectric properties compared to ternary and quaternary tetrahedrites produced with the same method,^54^ while tetrahedrites codoped with Ni, Zn, or cobalt on the Cu-site and Se or Te on the sulfur-site have also had their thermoelectric properties studied.^55−58^ Additionally, the modified polyol process was previously used to synthesize phase-pure tetrahedrite nanoparticles codoped with Zn on the Cu-site and Se on the sulfur-site, but Cu-site codoped nanomaterials were not synthesized.^41^ None of the aforementioned studies discussed the thermal stability or optical band gaps of codoped tetrahedrite materials, both of which are important material properties for thermoelectric and photovoltaic applications.

In this study, the modified polyol synthesis of quaternary single-doped tetrahedrite nanoparticles (Cu_11_M_1_Sb_4_S_13_, M = Zn, Fe, Ni, Mn, or Co) and quintenary tetrahedrite nanoparticles codoped on the Cu-site (Cu_11_M_0.5_N_0.5_Sb_4_S_13_, M, N = Zn, Fe, Ni, Mn, or Co) was undertaken. To the authors’ knowledge, synthesis of tetrahedrite codoped on the Cu-site by a solution-phase method or solution-phase synthesis of Mn-doped tetrahedrite has not been published previously. A comprehensive analysis of the impact of doping and codoping on the thermal stability of tetrahedrite nanoparticles was performed and the thermal stability of codoped nanoparticles was compared to the thermal stability of relevant single-doped nanomaterials. Additionally, a systematic examination of the optical properties for undoped, single-doped, and codoped tetrahedrite nanoparticles was undertaken. The optical absorption spectra and band gap energies for single-doped and codoped tetrahedrite nanoparticles were determined and compared for the first time. All tetrahedrite nanoparticles were characterized with X-ray powder diffraction and energy dispersive X-ray spectroscopy to ensure phase-purity and determine elemental composition. Thermogravimetric analysis was used to evaluate the thermal stability of the nanomaterials. Diffuse-reflectance vis–NIR spectroscopy investigated the optical behavior of the quaternary and quintenary tetrahedrite nanoparticles, and Tauc plots were used to determine the magnitude and character of the band gap. To facilitate the design of efficient and promising earth-abundant and nontoxic thermoelectric and photovoltaic materials for green energy applications, this research provides analysis of the impact of doping and codoping on the thermal and optical properties of tetrahedrite nanoparticles.

## 2 Experimental Section

### 2.1 Materials

The following reagents were precursors for the modified polyol synthesis of tetrahedrite nanoparticles and were used as received from Sigma-Aldrich Chemical Co.: antimony(III) acetate, (Sb(C_2_H_3_O_2_)_3_, ≥99.99%), copper(II) acetate monohydrate (Cu(C_2_H_3_O_2_)_2_·H_2_O, ≥98%), sulfur powder (99.98%), zinc(II) acetate (Zn(C_2_H_3_O_2_)_2_, 99.99%), iron(II) acetate (Fe(C_2_H_3_O_2_)_2_, 95%), nickel(II) acetate tetrahydrate (Ni(C_2_H_3_O_2_)_2_·4H_2_O, 98%), manganese(II) acetate (Mn(C_2_H_3_O_2_)_2_, 98%), and cobalt(II) acetate (Co(C_2_H_3_O_2_)_2_, 99.99%). Tetraethylene glycol (99%) from Alfa Aesar and ThermoScientific was the solvent, while sodium borohydride powder (NaBH_4_, ≥98.0%) from Sigma-Aldrich was used as the reducing agent. Anhydrous ethanol (200 proof, USP grade) from Pharmco-Aaper was utilized to isolate the product.

### 2.2 Synthetic Procedure

For the polyol synthesis of approximately 0.7 g of undoped tetrahedrite nanoparticles, the following reagents were added into a 250 mL round-bottom flask: Sb(C_2_H_3_O_2_)_3_ (1.7 mmol, 0.50 g), Cu(C_2_H_3_O_2_)_2_·H_2_O (5.0 mmol, 1.0 g), and sulfur (5.4 mmol, 0.17 g). The precursors were dispersed in 50 mL of tetraethylene glycol, creating a blue solution. The solution was sparged with N_2_ for 10 min with constant magnetic stirring. After sparging, the round-bottom flask was transferred to a reflux setup with a positive N_2_ atmosphere. Approximately 26 mmol (1.0 g) of NaBH_4_ was dispersed in 25 mL of tetraethylene glycol with the assistance of sonication and then slowly added to the precursor solution. Upon adding NaBH_4_, the color of the solution immediately changed from blue to dark brown and the temperature increased. Using an IKA RET control visc hot plate, the solution was heated at approximately 15° min^–1^ with constant magnetic stirring to 220 °C. The solution was held at 220 °C for 1 h and then allowed to cool to room temperature. The product was loaded into 50 mL centrifuge tubes and centrifuged at 4000 rpm for 10 min. The supernatant was discarded and the pellet was washed using ∼25 mL of absolute ethanol, sonicated, and centrifuged again. This process was repeated three times, and the resulting final products were dried overnight in a vacuum chamber. The tetrahedrite nanoparticle powder was dark brown in color.

To synthesize 0.7 g of single-doped tetrahedrite (Cu_11_MSb_4_S_13_, M = Zn, Fe, Ni, Mn, Co), 4.6 mmol (0.92 g) of Cu(C_2_H_3_O_2_)_2_·H_2_O was added, and 0.42 mmol of one of the following reagents was added as the dopant: Zn(C_2_H_3_O_2_)_2_ (0.077 g), Fe(C_2_H_3_O_2_)_2_ (0.075 g), Ni(C_2_H_3_O_2_)_2_·4H_2_O (0.10 g), Mn(C_2_H_3_O_2_)_2_ (0.071 g), or Co(C_2_H_3_O_2_)_2_ (0.073 g). The amount of Sb and sulfur precursor added was the same as stated for the synthesis of undoped tetrahedrite. To synthesize 0.7 g of codoped tetrahedrite (Cu_11_M_0.5_N_0.5_Sb_4_S_13_, M/N = Zn, Fe, Ni, Mn, Co) 4.6 mmol (0.92 g) of Cu(C_2_H_3_O_2_)_2_·2H_2_O was added alongside 0.21 mmol of two of the dopant salts and stoichiometric amounts of Sb and sulfur precursor. The synthesis and isolation procedures for doped tetrahedrite nanoparticles were as described previously. Single-doped and codoped tetrahedrite powders were brown in color, except for Cu_11_ZnSb_4_S_13_ sample, which was brick red.

### 2.3 Characterization Methods

The undoped, single-doped, and codoped tetrahedrite nanoparticles were characterized by X-ray diffraction (XRD), scanning electron microscopy (SEM), transmission electron microscopy (TEM), and energy dispersive X-ray spectroscopy (EDS) to obtain structural and compositional information. The thermal stability of the nanoparticles was studied using thermogravimetric analysis (TGA). Solid-state diffuse-reflectance vis–NIR spectroscopy was utilized to study the optical properties of the nanoparticles.

A Rigaku Miniflex II benchtop diffractometer was used to obtain nanoparticle powder XRD patterns. Data was collected over a 2θ range of 10° to 70° with Cu Kα radiation of 30 kV and 15 mA with a scan speed of 1° min^–1^ and a scan width of 0.03°. Lattice constants and grain sizes of the tetrahedrite products were calculated using the Rietveld refinement function of the PDXL2 software. PDF card #01-074-0270 was used to match the tetrahedrite phase.^29^

SEM and EDS characterization was completed using a JEOL JSM IT-200LA scanning electron microscope equipped with a JEOL JED-2300 Dry SDD EDS detector. EDS spectra were acquired using an electron beam with a 15 keV accelerating voltage. Tetrahedrite nanoparticle powders were analyzed at three or more separate locations to confirm sample homogeneity. Average elemental composition and associated standard deviations were calculated from EDS maps taken at each location. TEM images were collected with a Delong Low Voltage LVEM 25 microscope using the Zyla 5.5 Scientific CMOS camera under an electron beam with a 25 keV accelerating voltage. Samples were drop-cast from ethanol onto gold-coated carbon TEM grids (Electron Microscopy Sciences). Images were collected at several locations on each sample. Size distributions were determined through analysis using Fiji software for measurements.

TGA analysis of tetrahedrite nanoparticles was completed using a TA TGA Q500. Approximately 10 mg of nanoparticle powder was placed into an alumina sample pan, then heated at a rate of 10 °C min^–1^ with a sampling time of 2.00 s per point from room temperature to 1000 K. Heating was completed under a positive N_2_ atmosphere with a sample purge flow of 60 mL min^–1^. All samples were run at least twice to ensure reproducibility.

The optical properties of the tetrahedrite nanoparticles were investigated according to the procedure published by Jensen et al.^59^ Tetrahedrite nanoparticles were drop-cast from an ethanolic solution onto NIR-transparent quartz plates (Spectrocell) and a second clean quartz plate was placed on top, creating a sandwich sealed on the sides with parafilm. Solid-state diffuse-reflectance vis–NIR absorbance spectra were then collected using the integrating sphere of a PerkinElmer Lambda 950 spectrophotometer. A PMT detector using autogain and a slit width of 1.5 nm was the visible light detector, while a InGaAs detector with a gain of 10 and servo-controlled slit width was the near-infrared detector. Tetrahedrite nanoparticles were analyzed at the reflectance port of the integrating sphere over a range of 1800 to 400 nm in 3 nm increments. To autozero the spectrophotometer, two clean quartz plates were sandwiched together in front of a Spectralon reference.

The optical band gaps of the tetrahedrite nanoparticle samples were assessed using Tauc plots. The normalized absorbance (*A*) was utilized instead of the absorptivity (α) for the Tauc plots, as α could not be used because film thickness (*t*) was not quantified. Using *A* does not impact the linearity or *x*-intercept of the Tauc plots, as *A* is related to α through the relationship *A* = *t* × 2.303 × α. Therefore, the band gaps of the tetrahedrite nanoparticles were derived by fitting the linear region of (*Ah*ν)^*n*^ where *A* is the normalized absorbance, *h* is Planck’s constant, ν is the frequency, and *n* equals 2 or 1/2 for direct and indirect transitions, respectively.

### 2.4 General Safety Concerns

The synthetic procedures in this publication were performed at elevated temperatures using a high boiling point solvent under a positive N_2_ atmosphere. Glassware and heating equipment may reach temperatures of near or over 250 °C. Standard laboratory personal protective equipment should be worn at all times. Sulfur and hydrogen gases may be evolved during the reaction; therefore, a fume hood should be utilized. The safety data sheets for nontoxic metal salt precursors and elemental sulfur used should be reviewed before synthesis. Of particular note with regards to safety is the utilization of sodium borohydride (NaBH_4_) as a reducing agent. NaBH_4_ is highly reactive with water (Category 1) and upon contact releases flammable gases that may ignite when exposed to heat. Take extra precautions to limit exposure to water, and ensure that NaBH_4_ is not handled in close proximity to heat sources. NaBH_4_ is a Category 1B skin irritant and a Category 1 eye irritant that can cause severe burns upon contact with skin and eyes. Handle with care and ensure that proper eye protection and skin protection is utilized.

## 3 Results and Discussion

Undoped ternary (Cu_12_Sb_4_S_13_), single-doped quaternary (Cu_11_M_1_Sb_4_S_13_; M = Zn, Fe, Ni, Mn, or Co), and codoped quintenary (Cu_11_M_0.5_N_0.5_Sb_4_S_13_; M, N = Zn, Fe, Ni, Mn, or Co) nanoparticles were synthesized via the rapid, energy efficient modified polyol process. The phase-purity of the nanoparticles was analyzed using XRD with Rietveld refinements applied to ascertain lattice parameters and grain sizes of the tetrahedrite nanomaterials. SEM and TEM were utilized to observe surface morphology and confirm nanostructuring of the tetrahedrite particles, while EDS was used to obtain compositional data and investigate the distribution of constituent and dopant elements. The thermal stability of the undoped, single-doped, and codoped tetrahedrite nanoparticles was studied using TGA. Finally, vis–NIR spectroscopy was applied to discern the optical properties of the polyol-synthesized tetrahedrite nanoparticles, with the character and magnitude of the optical band gaps determined by Tauc plots. In this study, the effects of incorporating transition metal dopants on the structural, thermal, and optical properties of tetrahedrite nanoparticles synthesized by a modified polyol method are analyzed and discussed.

### 3.1 Structural and Compositional Characterization

**Figure 2:**
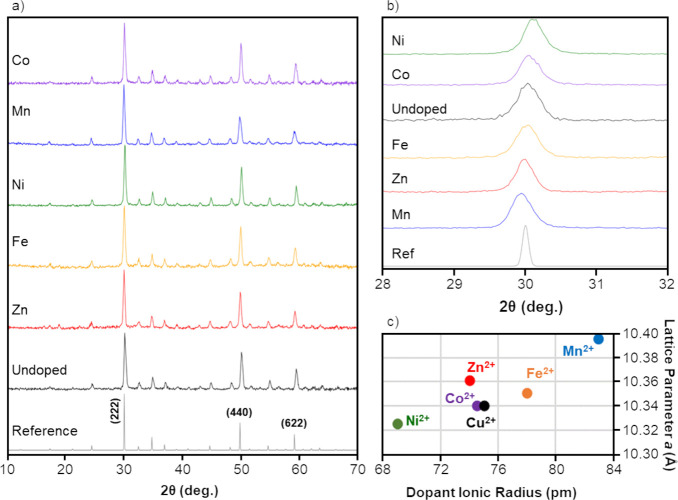
(a) Experimental X-ray powder diffraction patterns of undoped and single-doped tetrahedrite nanoparticles with the three dominant peaks indexed on the reference pattern (PDF Card #01-071-0270).^29^ Patterns are labeled according to the identity of the dopant for the formula Cu_11_M_1_Sb_4_S_13_, where M = Zn, Fe, Ni, Mn, or Co. (b) Region of pattern featuring the (222) peak selected to show dopant-dependent shifts in the peak position. (c) Calculated (222) lattice parameter for undoped and single-doped tetrahedrite nanoparticles graphed against the ionic radii of the respective dopants.^60^

Characterization of the structure of undoped and single-doped tetrahedrite nanoparticles showed large, pure phase tetrahedrite grains with shifts in the lattice parameter (*a*) due to doping. Figure 2a displays the experimental diffraction patterns of undoped (Cu_12_Sb_4_S_13_) and single-doped (Cu_11_M_1_Sb_4_S_13_; M = Zn, Fe, Ni, Mn, or Co) tetrahedrite nanoparticles alongside a reference pattern.^29^ All samples exhibited well-defined and symmetrical peaks that match in both position and relative intensity to the reference pattern with no extraneous peaks. This indicates that tetrahedrite nanomaterials are crystalline species with no preferred orientation observed. A close-up of the (222) peak (∼30° on the 2θ) is presented in Figure 2b and shows shifts in the (222) peak attributed to dopant incorporation. The (222) peak was shifted to lower values of 2θ in the Mn-, Fe-, and Zn-doped nanoparticles, while the (222) peak was shifted to higher values of 2θ in the Ni- and cobalt-doped nanoparticles. Similar dopant-dependent shifts in the experimental diffraction patterns for tetrahedrite have been observed in the literature.^36,37^ This shift is likely caused by changes in the lattice spacings induced by the incorporation of transition metal dopants with different ionic radii. Figure 2c plots the calculated lattice parameter (*a*) of the cubic unit cell for undoped and single-doped nanoparticles against the ionic radius of the dopant. All dopants are expected to possess a 2+ oxidation state, except for Fe, which may possess a 2+ or 3+ oxidation state.^32,36^ While dopants can be incorporated into Cu(I) or Cu(II) positions,^32,36^ the Cu(II) radius is selected for comparison in Figure 2c to mirror the anticipated oxidation state of most dopant species. Transition metal ions with smaller radii correlate to tetrahedrite nanoparticles with a smaller lattice parameter (*a*), except for the Zn-doped sample that has a higher lattice parameter than expected given that Zn(II) has a similar ionic radius to Cu(II). However, two studies have found an increased lattice parameter for Zn-doped tetrahedrite compared to the undoped sample, which is consistent with findings herein.^36,37^Table S1 shows the calculated lattice parameter (*a*) for the polyol-synthesized ternary and quaternary tetrahedrite nanoparticles as ranging from 10.32 Å (Ni-doped) to 10.40 Å (Mn-doped). Corresponding calculated grain sizes (grain size being defined as the diameter of a single tetrahedrite crystallite) range from 150 Å (Mn-doped) to 190 Å (Fe-doped), with an average of 170 ± 10 Å (Table S1). No correlation between the magnitude of the lattice parameter and grain size was observed. Rietveld refinement plots for undoped and single-doped tetrahedrite samples are shown in the Supporting Information in Figures S1–S6.

**Figure 3:**
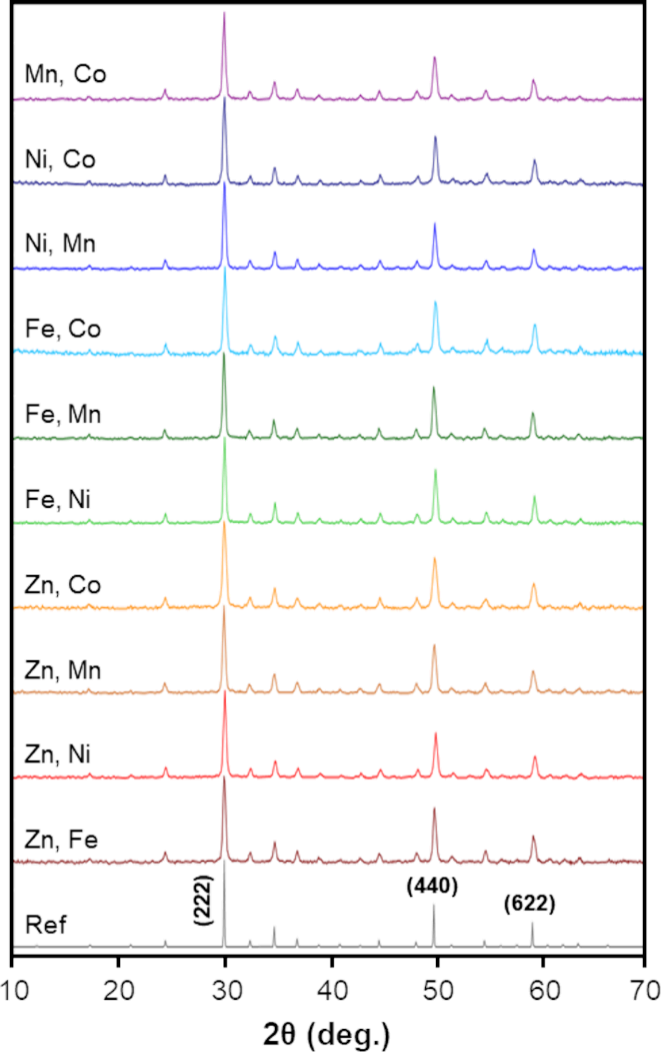
Experimental X-ray powder diffraction patterns for codoped tetrahedrite nanoparticles with the three dominant peaks indexed on the reference pattern (PDF Card #01-071-0270).^29^ Patterns are labeled according to the identity of dopants *M, N* for the formula Cu_11_M_0.5_N_0.5_Sb_4_S_13_, where M and N are Zn, Fe, Ni, Mn, or Co.

Analysis of the experimental diffraction patterns for the codoped tetrahedrite nanoparticles revealed that all codoped tetrahedrites are crystalline and phase-pure, displaying no preferred orientation. The experimental diffraction patterns for the codoped, quintenary tetrahedrite nanoparticles (Cu_11_M_0.5_N_0.5_Sb_4_S_13_; M, N = Zn, Fe, Ni, Mn, or Co) are shown in Figure 3 alongside a reference pattern.^29^ In the case of codoped samples, less shifting in peak position due to dopant ionic radii was observed. As shown in Table S1, the magnitude of the calculated lattice parameter (*a*) in the codoped tetrahedrites ranged from 10.34 Å (Ni_0.5_Co_0.5_ combination) to 10.37 Å (Fe_0.5_Mn_0.5_ combination), which is smaller than the range observed for the single-doped samples (10.32 Å–10.40 Å). The grain sizes of the codoped tetrahedrite nanoparticles ranged from 150 Å (Zn_0.5_Co_0.5_ combination) to 240 Å (Ni_0.5_Mn_0.5_ combination), with an average of 200 ± 30 Å.

**Figure 4:**
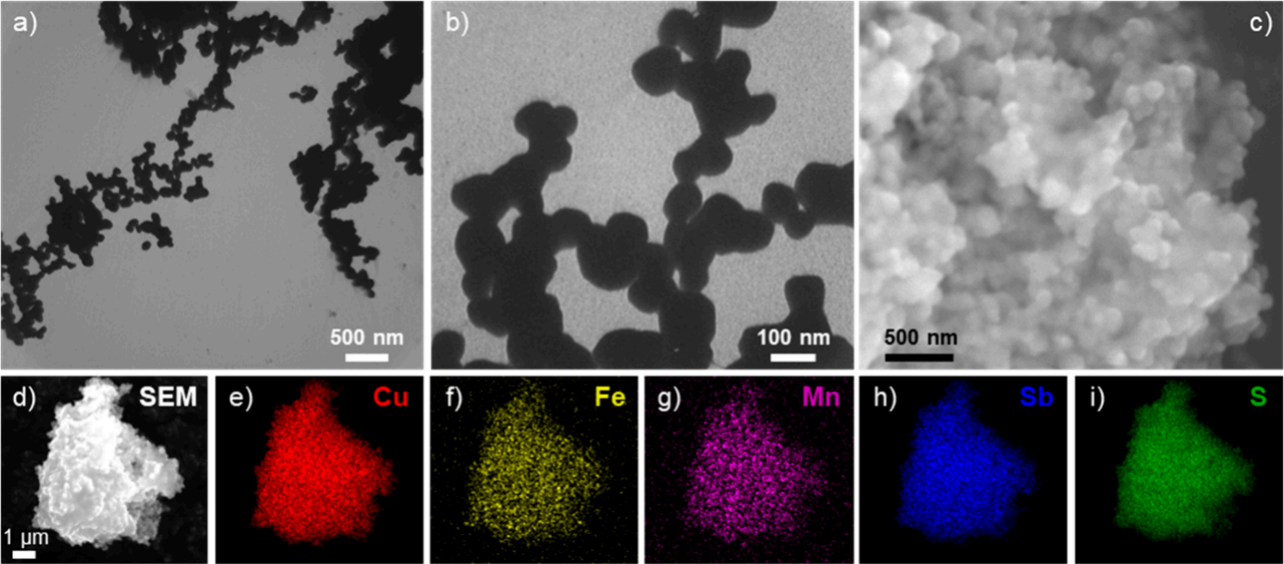
(a, b) Representative TEM images of the Cu_11_Fe_0.5_Mn_0.5_Sb_4_S_13_ sample showing individual nanoparticles with a diameter of 50–200 nm. (c) Representative SEM image of the Cu_11_Fe_0.5_Mn_0.5_Sb_4_S_13_ sample displaying clusters of nanoparticles. (d) SEM image and (e–i) associated elemental maps for the Cu_11_Fe_0.5_Mn_0.5_Sb_4_S_13_ tetrahedrite sample. The scale bar associated with (d) is the same for (e–i).

Representative electron microscopy images and corresponding elemental maps of codoped tetrahedrite nanoparticles with a target composition of Cu_11_Fe_0.5_Mn_0.5_Sb_4_S_13_ are shown in Figure 4. TEM images (Figure 4a,b, Figure S7) reveal that codoped tetrahedrite products are nanostructured, with size analysis (Supporting Information) finding that the diameter of the nanoparticles ranges from 50 to 200 nm (Figure S7). As Rietveld refinement analysis indicated grain sizes of ∼15–20 nm for tetrahedrite nanomaterials, the TEM and SEM imaging (Figure 4a–c) showed that tetrahedrite nanoparticles were polycrystalline and oblong in shape.

Elemental compositions for all tetrahedrite nanoparticles were within the compositional range of Cu_12–14.5_Sb_4–4.5_S_13_ that is commonly observed in natural and synthetic tetrahedrite materials.^24,39−41,61,62^ The elemental compositions determined by EDS mapping for all ternary undoped, quaternary single-doped, and quintenary codoped tetrahedrite nanoparticles are available in Table 1. An SEM image alongside EDS maps (Figure 4d–i) taken for the Cu_11_Fe_0.5_Mn_0.5_Sb_4_S_13_ codoped tetrahedrite nanomaterials indicates that constituent elements and dopants were distributed homogeneously throughout the sample, confirming that no amorphous impurities were found in the tetrahedrite nanomaterials. Previous research has shown that the modified polyol process fabricates phase-pure tetrahedrite nanoparticles with stoichiometric control and homogeneous elemental distribution.^39−41^ However, this is the first time that EDS analysis of tetrahedrite nanoparticles codoped on the Cu-site has been presented. Cu enrichment was observed for undoped tetrahedrite and was found to decrease with the incorporation of dopants. The undoped tetrahedrite had a composition of Cu_13.8±0.3_Sb_4.40±0.07_S_13.0±0.2_, which is within the acceptable range of tetrahedrite but is 15% Cu-enriched relative to the stoichiometric target of Cu = 12. Natural and synthetic tetrahedrite often displays a tendency for Cu enrichment because of vacancy sites throughout the lattice.^24,39−41,61,62^ In the modified polyol synthesis, Cu enrichment is hypothesized to be caused by sulfur volatilization or sulfur–solvent complexation throughout the synthesis.^41^ On average, single-doped samples displayed a Cu+M (copper and copper-site dopant) enrichment of 8%, while the codoped tetrahedrite nanoparticles had an average Cu+M+N (copper and copper-site codopants) enrichment of 7%. Most single-doped and codoped tetrahedrite nanoparticles displayed dopant enrichment, with only the Fe_0.5_Mn_0.5_ codoped nanoparticles having a dopant ratio (Mn = 0.41 ± 0.02) below the target amount (Mn = 0.5). Sb enrichment ranging from 3–12% above target value was consistently observed across all tetrahedrite nanoparticles. Like Cu enrichment, Cu-site dopant and Sb enrichment have been observed for tetrahedrite.^39−41^

**Table 1:** Elemental Composition of Tetrahedrite Nanoparticles^a^

Abbreviation	Target Composition	Cu	Dopant 1^b^	Dopant 2^c^	Sb	S
Undoped	Cu_12_Sb_4_S_13_	13.8 ± 0.3	-	-	4.40 ± 0.07	13.0 ± 0.2
Zn_1_	Cu_11_ZnSb_4_S_13_	11.9 ± 0.4	1.2 ± 0.1	-	4.25 ± 0.03	13.0 ± 0.5
Fe_1_	Cu_11_FeSb_4_S_13_	12.1 ± 0.1	0.97 ± 0.07	-	4.2 ± 0.1	13.00 ± 0.03
Ni_1_	Cu_11_NiSb_4_S_13_	11.2 ± 0.5	1.13 ± 0.02	-	4.14 ± 0.07	13.0 ± 0.5
Mn_1_	Cu_11_MnSb_4_S_13_	12.1 ± 0.2	1.20 ± 0.02	-	4.49 ± 0.03	13.0 ± 0.2
Co_1_	Cu_11_CoSb_4_S_13_	12.3 ± 0.2	1.06 ± 0.07	-	4.22 ± 0.07	13.0 ± 0.2
Zn_0.5_Fe_0.5_	Cu_11_Zn_0.5_Fe_0.5_Sb_4_S_13_	12.2 ± 0.5	0.50 ± 0.06	0.55 ± 0.04	4.25 ± 0.05	13.0 ± 0.5
Zn_0.5_Ni_0.5_	Cu_11_Zn_0.5_Ni_0.5_Sb_4_S_13_	11.7 ± 0.2	0.5 ± 0.1	0.54 ± 0.09	4.31 ± 0.05	13.0 ± 0.2
Zn_0.5_Mn_0.5_	Cu_11_Zn_0.5_Mn_0.5_Sb_4_S_13_	12.3 ± 0.4	0.62 ± 0.03	0.57 ± 0.02	4.41 ± 0.05	13.0 ± 0.5
Zn_0.5_Co_0.5_	Cu_11_Zn_0.5_Co_0.5_Sb_4_S_13_	12.2 ± 0.9	0.65 ± 0.06	0.62 ± 0.04	4.49 ± 0.08	13.0 ± 0.9
Fe_0.5_Ni_0.5_	Cu_11_Fe_0.5_Ni_0.5_Sb_4_S_13_	11.0 ± 0.3	0.53 ± 0.03	0.56 ± 0.07	4.37 ± 0.08	13.0 ± 0.3
Fe_0.5_Mn_0.5_	Cu_11_Fe_0.5_Mn_0.5_Sb_4_S_13_	11.4 ± 0.2	0.58 ± 0.02	0.41 ± 0.02	4.42 ± 0.02	13.0 ± 0.2
Fe_0.5_Co_0.5_	Cu_11_Fe_0.5_Co_0.5_Sb_4_S_13_	12.2 ± 0.3	0.51 ± 0.08	0.49 ± 0.08	4.21 ± 0.06	13.0 ± 0.2
Ni_0.5_Mn_0.5_	Cu_11_Ni_0.5_Mn_0.5_Sb_4_S_13_	11.79 ± 0.07	0.6 ± 0.1	0.55 ± 0.07	4.26 ± 0.07	13.0 ± 0.5
Ni_0.5_Co_0.5_	Cu_11_Ni_0.5_Co_0.5_Sb_4_S_13_	10.9 ± 0.6	0.56 ± 0.02	0.50 ± 0.03	4.27 ± 0.03	13.0 ± 0.7
Mn_0.5_Co_0.5_	Cu_11_Mn_0.5_Co_0.5_Sb_4_S_13_	11.7 ± 0.3	0.61 ± 0.01	0.5 ± 0.1	4.35 ± 0.03	13.0 ± 0.4

a Elemental composition determined via averaging three or more EDS maps. All ratios are normalized to *S* = 13.

b For codoped samples, “Dopant 1” is the first dopant listed in the abbreviation and target composition of the sample.

c For codoped samples, “Dopant 2” is the second dopant listed in the abbreviation and target composition of the sample.

The average elemental composition was Cu+M_13.0±0.4_Sb_4.3±0.1_S_13.0±0.2_ for single-doped, quaternary tetrahedrite nanoparticles and Cu+M+N_12.8±0.6_Sb_4.33±0.09_S_13±0.2_ for codoped, quintenary tetrahedrite nanoparticles. The low standard deviations in the EDS data collected at different locations throughout the sample confirm constituent elements and dopants were distributed homogeneously throughout the samples, in agreement with EDS maps. For the Cu, Sb, and sulfur ratios of codoped nanoparticles, the average relative standard deviation was below 3%. When including dopants, the average relative standard deviation increased to approximately 6%; however, the absolute error remained low, indicating that the increase in average relative standard deviation can be attributed to the low target value for the dopant ratio. Elemental analysis of multinary tetrahedrite nanomaterials doped on the Cu-site showcases the robustness and flexibility of the modified polyol method, producing 0.7 g of high-quality nanoparticles per synthesis.

### 3.2 Thermal Properties

**Figure 5:**
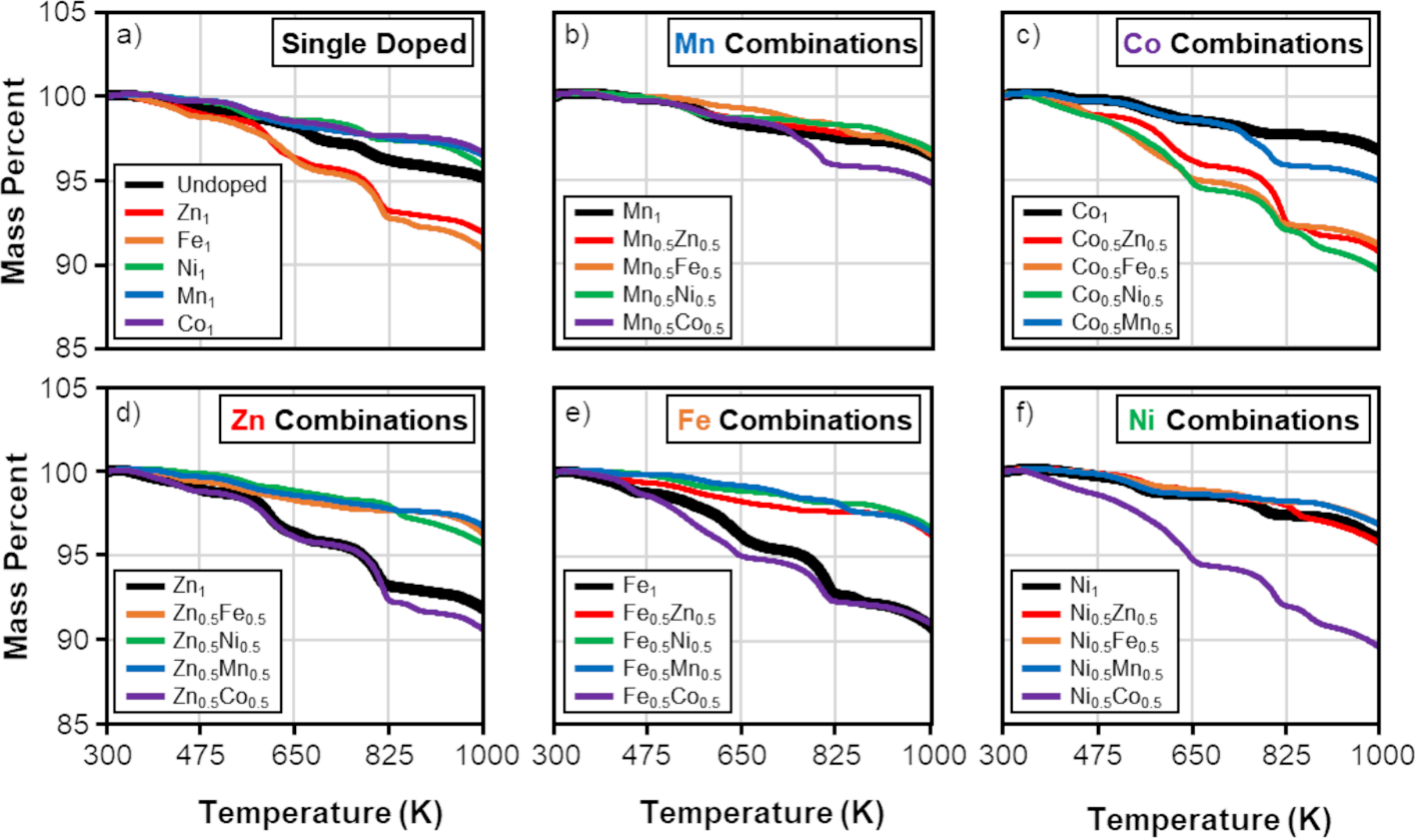
Thermal gravimetric analysis curves for (a) undoped (Cu_12_Sb_4_S_13_) and single-doped (Cu_11_M_1_Sb_4_S_13_; M = Zn, Fe, Ni, Mn, Co) tetrahedrite samples as well as thermal gravimetric analysis curves for the (b–f) codoped (Cu_11_M_0.5_N_0.5_Sb_4_S_13_; M, N = Zn, Fe, Ni, Mn, Co) combinations. Co-doped combinations are labeled such that the Zn_0.5_Fe_0.5_ sample is the same as the Fe_0.5_Zn_0.5_ sample.

Through TGA characterization of undoped, single-doped, and codoped tetrahedrite nanoparticles, doping with certain transition metals was identified to improve thermal stability. Figure 5a displays the thermogravimetric curves for the undoped (Cu_12_Sb_4_S_13_) and single-doped (Cu_11_M_1_Sb_4_S_13_, M = Zn, Fe, Ni, Mn, or Co) tetrahedrite nanoparticles. Between room temperature and 500 K, undoped and single-doped tetrahedrite nanoparticles experienced a steady mass loss of ∼1–2%, likely due to decomposition of residual solvent.^41,59^ At higher temperatures, all samples displayed two regions of steeper mass loss at approximately 600–675 K and 800–825 K. The mass loss between 600 and 675 K was previously observed for polyol-synthesized undoped and transition metal doped tetrahedrite but is not observed in solid-state synthesized bulk tetrahedrite.^41^ Therefore, it is thought that this mass loss is the result of sulfur volatilization engendered by nanostructuring. In the literature, mass loss at around 800 K was shown to correspond with an endothermic transition resulting from either a phase transformation from Cu_12_Sb_4_S_13_ to Cu-rich pseudotetrahedrite (Cu_3_SbS_3_) or decomposition into CuSbS_2_ and Cu_2–*x*_S phases, both with associated sulfur volatilization.^35,36^ Overall, single-doped nanoparticles retained >91% of their starting mass at 1000 K. However, the Zn_1_ (∼92% starting mass at 1000 K) and Fe_1_ (∼91% at 1000 K) tetrahedrite nanoparticles were less stable than the undoped nanoparticles (∼95% at 1000 K), while the Ni_1_, Mn_1_, and Co_1_ nanoparticles (all >96% at 1000 K) showed increased thermal stability. It is posited that an increase in thermal stability is due to the stability of dopant–sulfur interactions compared to Cu–sulfur interactions. Future work will look to probe observed differences in thermal stabilities using XPS methods.

There are a few studies in the literature that compare the thermal stability of undoped bulk tetrahedrite to transition metal doped bulk tetrahedrite. However, the results are inconsistent, which is perhaps due to differing synthetic or densification methods. For example, Pi et al. systematically studied the thermal stability of bulk transition metal doped tetrahedrite (Cu_11_TrSb_4_S_13_, Tr = Cu, Zn, Mn, Fe, Ni, or Co) and found that all bulk tetrahedrite samples retained >96% of their starting mass at 1000 K.^37^ In their study, the Fe-doped bulk tetrahedrite sample had the lowest remaining mass at 1000 K (∼96.5%), which is consistent with the findings in this study. However, all doped bulk tetrahedrite samples in the Pi et al. study all displayed decreased stability compared to the undoped bulk material.^37^ In contrast, other thermal stability studies such as those performed by Barbier et al. and Tippireddy et al. using TGA to analyze bulk tetrahedrite doped with transition metals showed increased thermal stability in transition metal doped bulk tetrahedrite samples,^35,36^ mirroring results from this study. In particular, Barbier et al. discovered that a Ni-doped tetrahedrite sample (Cu_11_NiSb_4_S_13_) retained ∼98.5% of starting mass at 925 K,^35^ while Tippireddy et al. observed that a Mn-doped bulk tetrahedrite sample (Cu_11.5_Mn_0.5_Sb_4_S_13_) retained 97.5% of starting mass at 1000 K.^36^ Both transition metal doped bulk samples displayed increased thermal stability relative to the undoped bulk tetrahedrite sample synthesized by Barbier et al. at the maximum temperature (∼96.5% of starting mass at 925 K).^35,36^ Previous research also investigated the effect of doping on the thermal stability of polyol-synthesized tetrahedrite and famatinite (Cu_3_SbS_4_) nanomaterials.^41,59^ TGA combined with differential scanning calorimetry showed that Cu-site doping with first-row transition metals stabilized tetrahedrite,^41^ while all these dopants except for Fe improved famatinite thermal stability.^59^ Those findings were further confirmed by the work herein, showing that the doping helps to compensate for lower stability associated with nanostructuring.

This is the first study to characterize tetrahedrite codoped on the Cu-site with thermal analysis methods, finding improved thermal stability for codoped tetrahedrite (Cu_11_M_0.5_N_0.5_Sb_4_S_13_; M, N = Zn, Fe, Ni, Mn, or Co) nanoparticles relative to undoped and single-doped samples (Figures 5b–f). The TGA curves for codoped nanoparticles are similar to the curves of the single-doped samples, with an initial gradual mass loss from room temperature to 500 K followed by two steeper transitions around 600 and 800 K. Figure 5b contains data for the Mn combinations, revealing that the Mn_0.5_Zn_0.5_, Mn_0.5_Fe_0.5_, and Mn_0.5_Ni_0.5_ codoped samples retained slightly more mass at 1000 K (all ∼97%) and displayed increased thermal stability relative to the Mn_1_ single-doped nanoparticles (96.5% starting mass at 1000 K). This shows codoping with Mn and one of the other dopants increases the thermal stability of tetrahedrite nanomaterial. In contrast, the Mn_0.5_Co_0.5_ sample (∼95% starting mass at 1000 K) was less stable than the Mn_1_ single-doped sample. Co-doping with cobalt was concluded to decrease stability, as shown by Figure 5c. The Co_0.5_Ni_0.5_, Co_0.5_Zn_0.5_, and Co_0.5_Fe_0.5_ samples retained ∼90% starting mass at 1000 K, exhibiting lower thermal stability relative to the Co_1_ single-doped sample (96.5% starting mass at 1000 K). The data shown for the Zn combinations (Figure 5d), Fe combinations (Figure 5e), and Ni combinations (Figure 5f) contained these trends observed in the Mn and cobalt combinations. Importantly, combinations of Zn or Fe with dopants other than cobalt displayed significantly improved thermal stability relative to Zn_1_ or Fe_1_ single-doped nanoparticles. The Zn_0.5_Fe_0.5_, Zn_0.5_Ni_0.5_, Zn_0.5_Mn_0.5_, Fe_0.5_Ni_0.5_, and Fe_0.5_Mn_0.5_ combinations all retained >97% of starting mass, more than the 92% and 91% mass retained at 1000 K for the Zn_1_ and Fe_1_ single-doped nanomaterials, respectively. As all codoped combinations except those containing cobalt retained ∼97% or more of their starting mass at 1000 K, they exhibit higher thermal stability than the undoped (∼95%) and single-doped tetrahedrite nanoparticles (∼91%–96%) and comparable thermal stability to bulk tetrahedrite material from the literature.^35−37^ As with the single-doped samples, changes in stability among codoped samples are thought to be caused by variation in dopant–sulfur interactions. Nanostructured materials display improved thermoelectric and photovoltaic performance;^4−7,39−45^ therefore, it is advantageous for high temperature applications that codoping with Zn, Fe, Ni, or Mn on the Cu-site generally counteracts the decrease in thermal stability from nanostructuring.

### 3.3 Optical Properties

**Figure 6:**
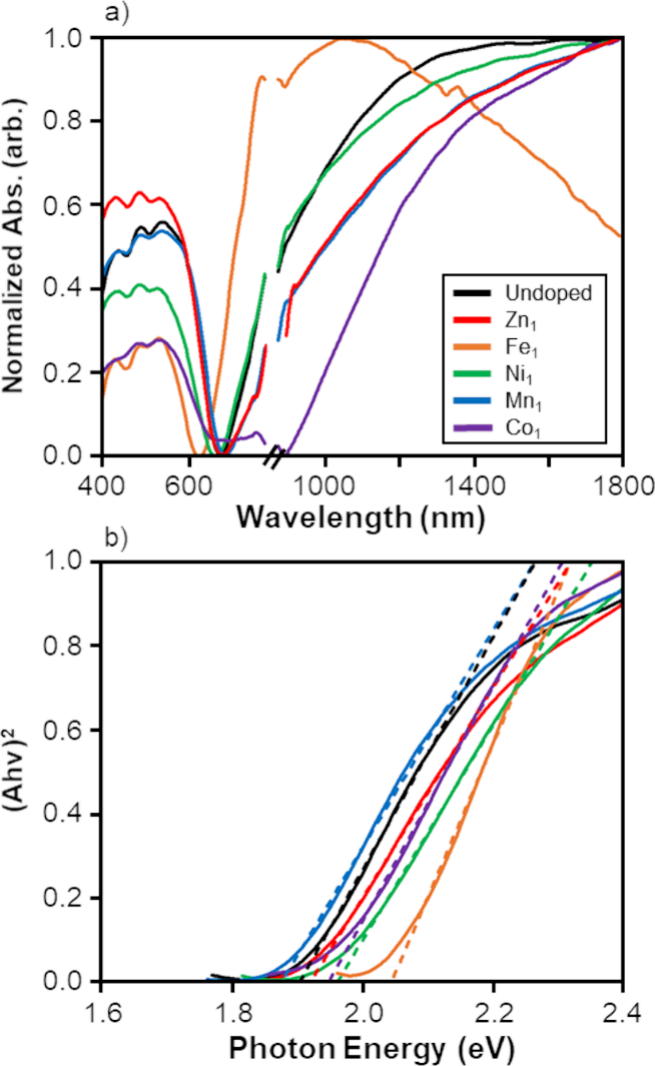
(a) Optical absorbance and (b) Tauc plot of (*Ah*ν)^2^ for undoped (Cu_12_Sb_4_S_13_) and single-doped (Cu_11_M_1_Sb_4_S_13_; M = Zn, Fe, Ni, Mn, or Co) tetrahedrite nanoparticles. In the optical spectra, the break in the *x*-axis from 785 to 875 nm is due to a detector changeover. The *x*-intercept of the linear fits for the Tauc plots in (b) yields the magnitude of the direct optical band gap (Table S2).

For the first time, optical characterization of doped and codoped tetrahedrite nanoparticles was undertaken, revealing that doping slightly alters the optical properties of tetrahedrite nanoparticles. The raw absorbance (Figure S8) and normalized absorbance (Figure 6a) spectra for the undoped and single-doped tetrahedrite nanoparticles displayed continuous absorbance from 400–1800 nm. A distinct, intense absorbance band was observed in both the visible light region and near-infrared region, respectively. The near-IR band is characterized by a steady increase in absorbance from 800–1800 nm in the spectra of all samples except the Fe_1_ single-doped sample. The Fe_1_ single-doped nanoparticle spectrum, which was generally blue-shifted relative to the undoped nanoparticle spectrum, displays a less broad near-IR band with a maximum (∼1100 nm), which was not observed for the undoped and other single-doped nanoparticles. For undoped and single-doped tetrahedrite nanoparticles, there was more variance seen in the shape and position of the near-IR band than seen in the visible light band.

The intense absorbance band in the near-IR region is a significant feature in the optical spectra of the polyol-synthesized tetrahedrite nanoparticles. While absorbance in both the visible and near-IR region was observed for tetrahedrite nanoparticles from the literature characterized by both solution-phase and thin-film optical spectroscopy methods, tetrahedrite nanoparticles usually display significantly weaker absorption in the near-IR region than the visible light region.^23,26,27,46−49^ Van Embden et al. synthesized nanoparticles ranging from 5–15 nm in diameter, and attributed increases in near-IR absorbance to decreased nanoparticle diameter.^46^ According to Rietveld refinement, the average grain size for polyol-synthesized tetrahedrite nanoparticles was 17 nm, similar to the Van Embden nanocrystals showing near-IR absorbance. Van Embden et al. also posited that near-IR absorbance could be the result of localized surface plasmon resonance behavior such as that exhibited by Cu_2–*x*_S nanocrystals, but quickly discarded this hypothesis after characterizing nanoparticles in solvents with different refractive indices.^46^ Ultimately, studies by both Prem Kumar et al. and Van Embden et al. attributed the presence of the near-IR absorbance band to free carrier absorption driven by a high density of midband defect states. This may explain the intense near-IR band also observed in the optical spectra of the polyol-synthesized tetrahedrite nanoparticles.^26,46^ The presence of a strong near-IR band increases the photovoltaic utility of the material, as it widens the range of photons that can be absorbed by the material.

Undoped and single-doped polyol-synthesized tetrahedrite nanoparticles were found to be direct band gap materials, as shown by consistent linear behavior in the (*Ah*ν)^2^ Tauc plots (Figure 6b) for all samples. The undoped tetrahedrite nanoparticles possessed a band gap of 1.90 eV. The Fe_1_ doped nanoparticles had the most blue-shifted band gap (2.04 eV), while the Mn_1_ doped nanoparticles had the most red-shifted band gap (1.88 eV). The band gap of the tetrahedrite nanoparticles is approximated by the position of the minimum between 600 to 700 nm in the tetrahedrite optical spectra (Figure 6a). The 1.95 eV band gap of the Co_1_ sample corresponds to a steep decline in the visible region of the optical spectrum (Figure 6a) at ∼600 nm prior to a small shoulder rather than the spectral minimum at ∼900 nm. A minimum at 900 nm would correspond to a red-shifted band gap closer to the optimal 1.2–1.5 eV range for photovoltaics.^8−10^ For this reason, additional investigation into the optical properties of cobalt-doped tetrahedrite nanoparticles is warranted. A summary of the band gap values and linear fits for undoped and single-doped nanoparticles is available in Table S2.

The polyol-synthesized undoped and single-doped tetrahedrite nanoparticles had direct band gaps ranging from 1.88–2.04 eV. Van Embden et al. were the first to characterize the optical band gap of tetrahedrite nanoparticles, finding a band gap of 1.7 eV for nanoparticles with a 9 nm diameter.^46^ In a later study, Ramasamy et al. obtained an experimental direct band gap of 1.7 eV for tetrahedrite nanoparticles and derived a band gap of 1.62 eV using density functional theory calculations.^23^ Chen et al. then observed a quantum confinement effect in tetrahedrite nanoparticles, discerning that the band gap increased from 1.89 to 2.40 eV as the nanocrystal diameter decreased from 15.9 to 2.2 nm.^48^ Rietveld refinement calculations predicted that individual polyol-synthesized tetrahedrite grains range in size from 14–20 nm, and optical characterization shows that polyol-synthesized nanomaterials possessed band gaps similar to the 15.9 nm nanoparticles characterized by Chen et al.^48^ Finally, Alqahtani et al. studied the impact of Zn incorporation on the band gap energy of tetrahedrite thin films, finding a band gap increase of 0.03 eV for the Cu_11_Zn_1_Sb_4_S_13_ sample relative to the undoped sample.^38^ A dopant-dependent shift in the band gap of a comparable amount was observed in the polyol-synthesized Zn-doped tetrahedrite, where the band gap increased from 1.90 eV (Cu_12_Sb_4_S_13_) by 0.02 to 1.92 eV (Cu_11_Zn_1_Sb_4_S_13_).^38^ Shifts of a similar magnitude in the band gap energies were observed for different dopants, and all single-doped tetrahedrite nanoparticles had band gap values within 0.14 eV of the undoped nanoparticles. In summary, the undoped and single-doped tetrahedrite nanoparticles studied herein displayed direct band gaps within the range reported in the literature (1.5–2.0 eV),^23,26,27,46−49^ while the incorporation of transition metal dopants Zn, Fe, Mn, Ni, or Co onto the Cu-site of tetrahedrite minimally altered the optical properties of the material.

**Figure 7:**
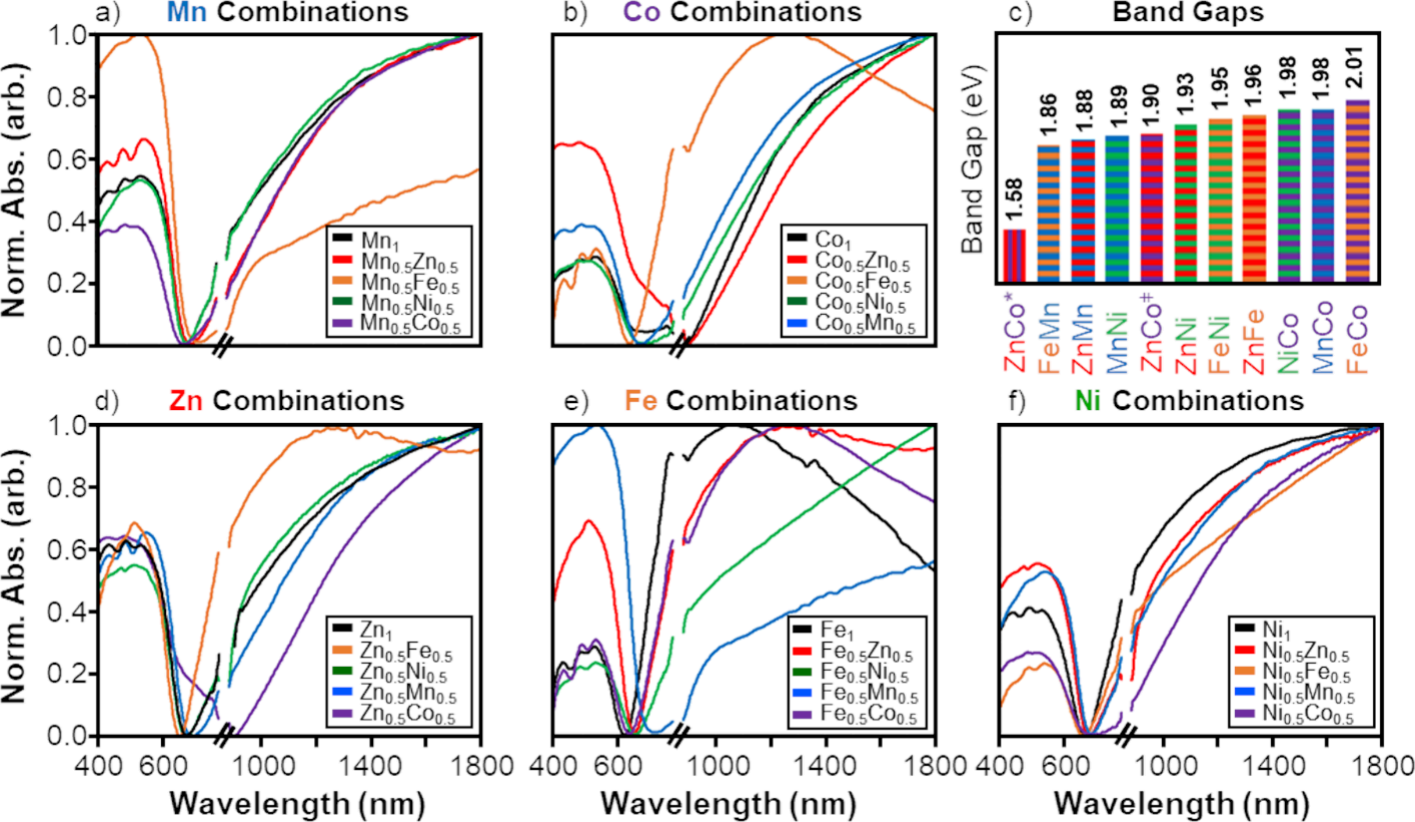
Vis–NIR absorbance of the (a) Mn, (b) Co, (d) Zn, (e) Fe, and (f) Ni codoped (Cu_11_M_0.5_N_0.5_Sb_4_S_13_; M, N = Zn, Fe, Ni, Mn, Co) combinations alongside the relevant single-doped sample. (c) Bar graph displaying the magnitude of the direct band gap for each codoped tetrahedrite combination. In the optical spectra, the break in the *x*-axis from 785 to 875 nm is due to a detector changeover. In (c), two band gaps for the Zn_0.5_Co_0.5_ sample are noted using ZnCo* and ZnCo^⧧^, respectively, as a result of the presence of two linear regions in the Tauc plot of *Ah*ν^2^.

For all codoped combinations, the impact of codoping on the optical properties of tetrahedrite nanoparticles was systematically analyzed with normalized absorbance as shown in Figure 7a,b,d–f. Absorbance bands in both the visible light and near-IR regions were observed in all codoped nanoparticles alongside a minimum ranging from 600–700 nm. The optical spectra of Mn codoped combinations (Figure 7a) except the Mn_0.5_Fe_0.5_ combination were similar in shape to the Mn_1_ single-doped nanoparticle spectra, exhibiting more relative absorbance in the near-IR region than in the visible light region. Other combinations with Fe such as the Co_0.5_Fe_0.5_ and Zn_0.5_Fe_0.5_ also behaved uniquely, as the optical spectra of both samples were blue-shifted relative to other combinations and featured distinct peaks in the near-IR range around 1200 nm, similar to what was observed for the Fe_1_ single-doped sample. Apart from the aforementioned combinations with Fe, the optical spectra of the cobalt codoped combinations (Figure 7b), Zn codoped combinations (Figure 7d), and Ni codoped combinations (Figure 7f) all demonstrated similar shape to the single-doped and Mn codoped nanoparticle spectra. This is in contrast to the plot of all Fe combinations (Figure 7e), where more varied behavior was displayed. Finally, all Fe codoped combinations and Ni codoped combinations had optical spectra that were red-shifted in the near-IR region relative to the Fe_1_ or Ni_1_ single-doped sample, respectively. Overall, except for Mn_0.5_Fe_0.5_, Co_0.5_Fe_0.5_, and Zn_0.5_Fe_0.5_ combinations, the optical spectra of the codoped nanoparticles were similar to those found for the single-doped tetrahedrite nanomaterials.

As seen in Figure 7c, the magnitude of the direct band gaps found for codoped tetrahedrite nanoparticles ranged from 1.85 eV (Fe_0.5_Mn_0.5_) to 2.01 eV (Fe_0.5_Co_0.5_), except for the Zn_0.5_Co_0.5_ nanoparticles. Tauc plots of (*Ah*ν)^2^ for the Mn, Co, Zn, Fe, and Ni codoped combinations are displayed in Figure S9a–e respectively, with associated band gap values and linear fits available in Table S3. The range of the direct band gap values for most codoped combinations were similar to the range found for the undoped and single-doped nanoparticles within this study and were within the range given by the literature (1.5–2.0 eV).^23,26,27,46−49^ Uniquely, the Tauc plot for the Zn_0.5_Co_0.5_ codoped combination (Figure S9b) had two linear fits which resulted in two direct band gaps of 1.58 and 1.90 eV, respectively, being determined. The higher energy band gap is associated with a shoulder in the optical spectrum (Figure 7b) around 650 nm that was also present in the Co_1_ spectrum, while the lower energy band gap is associated with the spectral minimum at ∼900 nm. A band gap energy of 1.58 eV is a significant red-shift from the other tetrahedrite nanoparticles and is much closer to the ideal range for photovoltaic application (1.2–1.5 eV);^8−10^ therefore, further investigation of the Zn_0.5_Co_0.5_ combination is an avenue for future work. In all other cases, the introduction of codopants did not significantly change the optical properties of the material.

### 3.5 Quintenary Grains

**Figure 8:**
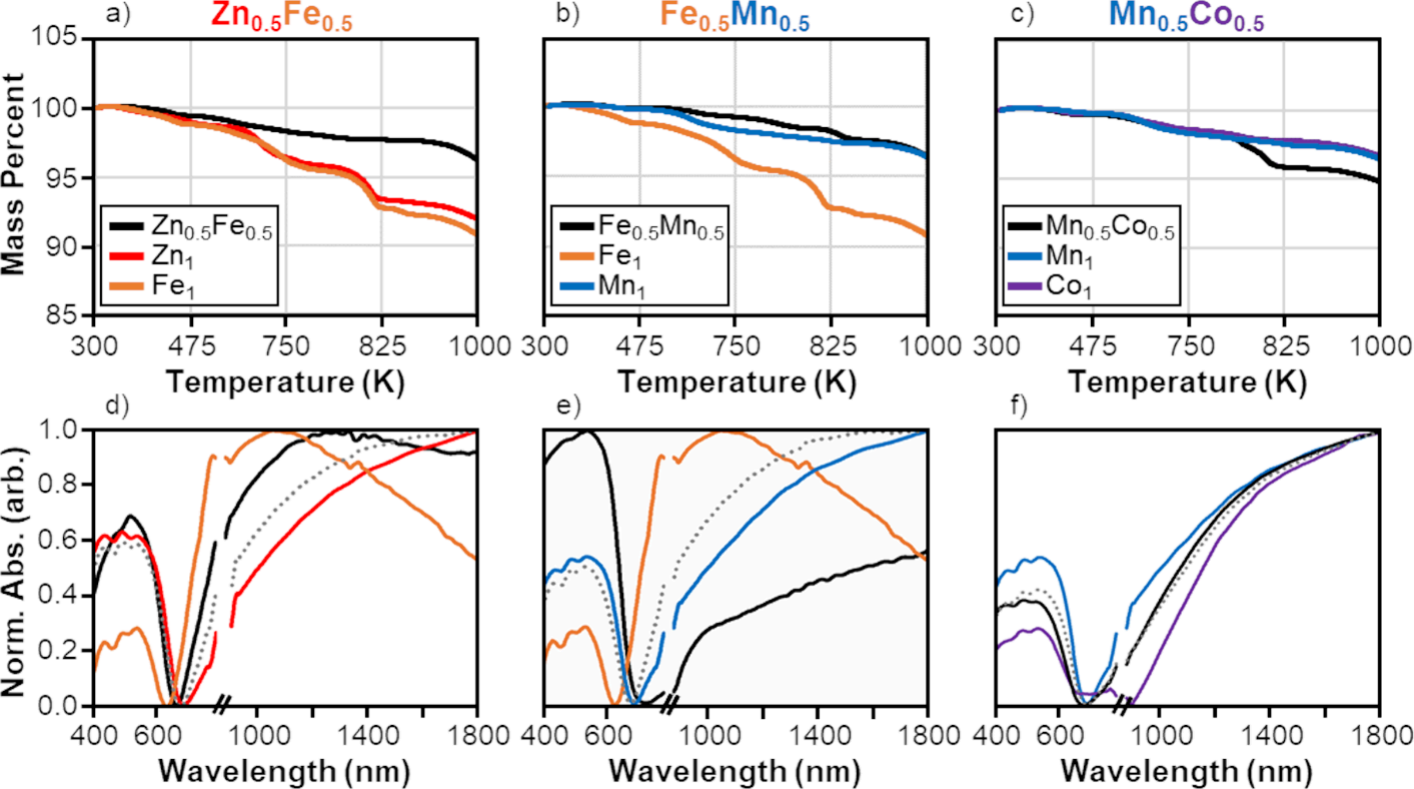
TGA curves of the (a) Zn_0.5_Fe_0.5_, (b) Fe_0.5_Mn_0.5_, and (c) Mn_0.5_Co_0.5_ codoped samples alongside the TGA curves of the two relevant single-doped nanoparticles. Optical spectra of the (d) Zn_0.5_Fe_0.5_, (e) Fe_0.5_Mn_0.5_, and (f) Mn_0.5_Co_0_ codoped nanoparticles alongside the optical spectra of the two relevant single-doped nanoparticles. Included is a dashed gray line showing the normalized average raw absorbance of the two single-doped nanoparticles.

Thermal and optical analysis suggested that the codoped tetrahedrite nanoparticles were true quintenary grains (Cu_11_M_0.5_N_0.5_Sb_4_S_13_), rather than a blend of two quaternary grains mixed in the same sample (Cu_11_M_1_Sb_4_S_13_ and Cu_11_N_1_Sb_4_S_13_). To support this finding, thermal data in Figure 8a–c displays the thermogravimetric curves of three codoped nanoparticles: the Zn_0.5_Fe_0.5_ sample (Figure 8a), Fe_0.5_Mn_0.5_ sample (Figure 8b), and the Mn_0.5_Co_0.5_ sample (Figure 8c). Included in each graph are the thermal gravimetric curves for the relevant single-doped samples. In all cases, the thermal gravimetric curves of the codoped nanoparticles were distinct from those of the single-doped nanoparticles. For a sample that is a blend of quaternary grains, it may be expected that the thermogravimetric curve would either be the average of the single-doped curves or display lower overall stability than both single-doped curves because of induced melting across the grain boundaries.

Copolymers provide a relevant comparison that help us understand why codoped tetrahedrite may have properties that are not the sum of the individual doped samples. There is often a difference in the glass transition temperatures between miscible polymer blends and copolymer materials of the same monomer components. For the miscible polymer blend that is 50% monomer A and 50% monomer B, the blend glass transition temperature usually follows the linear-addition law, meaning that it would be the weighted average of the monomer glass transition temperatures.^63,64^ However, the glass transition temperature for copolymer AB will not be the weighted average of the glass transition temperatures of monomer “A” and monomer “B” but may be higher, lower, or a non-mean value in between.^63,64^ This copolymer case is closer to what is observed for the selected codoped nanomaterials in Figure 8a–c and all other codoped combinations (Figure S10) because none of them display a thermal gravimetric curve that is the average of relevant single-doped curves.

Additional support for the existence of quintenary grains comes from comparing the optical properties of the codoped samples to the single-doped samples. Figure 8d–f shows the normalized absorbance for the Zn_0.5_Fe_0.5_ sample (Figure 8d), Fe_0.5_Mn_0.5_ sample (Figure 8e), and the Mn_0.5_Co_0.5_ sample (Figure 8f) alongside the optical spectra of relevant single-doped samples. For a blend of two nonreactive reagents, absorbance acts in an additive manner such that the total absorbance of blend AB represents the weighted addition of the absorbance of reagent A and reagent B.^65^ The dashed gray lines in Figure 8d–f represent the addition of the raw absorbances from each single-doped sample that is then normalized. For the selected codoped samples, the absorbance of the codoped sample was distinct from the weighted average (gray dashed line in Figure 8d–f). This was consistently observed in Figure S11, which displays the optical spectra of all other codoped nanoparticle combinations plotted alongside the optical spectra of relevant single-doped samples. Additionally, the band gap energies of the codoped combinations (Table S3) were never directly between band gap energies of relevant single-doped nanoparticles (Table S2). Therefore, it can be inferred that the codoped tetrahedrite nanoparticles were true quintenary grains instead of a blend of quaternary grains.

## 4 Conclusion

In this study, a modified polyol process was utilized to synthesize undoped (Cu_12_Sb_4_S_13_), single-doped (Cu_11_M_1_Sb_4_S_13_, M = Zn, Fe, Ni, Mn, or Co), and codoped (Cu_11_M_0.5_N_0.5_Sb_4_S_13_, M, N = Zn, Fe, Ni, Mn, or Co) tetrahedrite nanoparticles. This was the first time that quintenary tetrahedrite codoped on the Cu-site was synthesized by a solution-phase method. All tetrahedrite nanoparticles were phase-pure by XRD, with EDS analysis confirming that the undoped, single-doped, and codoped tetrahedrite nanoparticles were synthesized with a high degree of stoichiometric control. Furthermore, EDS showed that doping diminishes Cu enrichment in polyol-synthesized tetrahedrite nanoparticles. The thermal and optical properties of the undoped, single-doped, and codoped tetrahedrite nanoparticles were analyzed using TGA and vis–NIR methods, and Tauc plots were used to determine the character and magnitude of the band gap. In this study, the thermal stabilities were systematically investigated for Cu-site codoped tetrahedrite nanoparticles and a methodical analysis of the optical properties for single-doped and codoped tetrahedrite nanoparticles was undertaken, both for the first time. Finally, thermal and optical data suggested codoped nanoparticles consisted of true quintenary grains instead of a blend of quaternary grains.

In most cases, doping and codoping was found to increase the thermal stability of tetrahedrite nanoparticles. Quaternary Ni_1_, Mn_1_, and Co_1_ nanoparticles displayed improved thermal stability relative to undoped nanoparticles, retaining >95% of their starting mass at 1000 K, while all codoped tetrahedrite nanoparticles retained 90% or more of their starting mass at 1000 K. Generally, codoping with Mn, Fe, Ni, and Zn increased thermal stability, while codoping with cobalt decreased thermal stability. Specifically, the Zn_0.5_Fe_0.5_, Zn_0.5_Mn_0.5_, Fe_0.5_Ni_0.5_, Fe_0.5_Mn_0.5_, and Ni_0.5_Mn_0.5_ codoped nanoparticles were the most stable (<4% mass loss), showing increased thermal stability relative to all undoped and single-doped nanoparticles. Undoped, single-doped, and codoped tetrahedrite nanoparticles exhibited similar optical behavior, displaying strong, continuous absorbance from 400–1800 nm. Tetrahedrite nanomaterial spectra possessed broad absorbance bands in the visible and near-IR regions and a minimum between 600 and 700 nm, which estimates the magnitude of the optical band gap. The undoped tetrahedrite nanoparticles had a direct band gap of 1.90 eV, while single-doped and codoped tetrahedrite nanoparticles possessed direct band gaps ranging from 1.88 to 2.04 eV. In conclusion, dopant or codopant incorporation minimally impacted the optical properties and magnitude of the band gap in tetrahedrite nanoparticles.

Nanostructured materials offer considerable advantages for thermoelectric applications.^4−7^ The increased thermal stability of the quintenary codoped nanoparticles can balance the loss in stability inherent to nanostructured materials, which is beneficial for high-temperature applications. Therefore, investigating the thermoelectric performance of polyol-synthesized codoped tetrahedrite nanoparticles is an avenue for future investigation. Research would test if the more thermally stable codoped tetrahedrite nanoparticles display comparable or improved thermoelectric properties relative to undoped and single-doped tetrahedrite nanoparticles. Another key finding is that doping or codoping tetrahedrite nanoparticles can improve thermal stability without significantly altering the optical properties of the material. For future work investigating increasing photovoltaic performance and fabricating solar cells, the processability of tetrahedrite nanoparticles will be investigated via surface functionalization. Developing high-performance thermoelectric and photovoltaic materials that are safe, sustainable, and affordable is an important step toward implementing long-term energy solutions. Understanding the thermal and optical properties of quaternary and quintenary tetrahedrite nanoparticles with improved thermal stability for high temperature applications and broad optical absorption that can be tailored may provide a route for the future development of effective green-energy technologies.

## Supporting Information Available

Rietveld refinement plots and data, TEM and associated size distribution analysis for the Fe_0.5_Mn_0.5_ codoped sample, raw absorbance for undoped and single-doped nanoparticles, Tauc plots for codoped combinations, tables summarizing band gaps and Tauc plot linear fits for all samples, and thermal and optical data for codoped nanoparticles plotted against relevant single-doped nanoparticles (PDF)
