# Supplementary material for: Multinary Tetrahedrite (Cu12–x–yMxNySb4S13) Nanoparticles: Tailoring Thermal and Optical Properties with Copper-Site Dopants
Source: Chem Mater. 2024 Mar 25;36(7):3246–58. doi: 10.1021/acs.chemmater.3c03110 (PMC11007862; doi:10.1021/acs.chemmater.3c03110)
Supplement: Supplementary file 1 — cm3c03110_si_001.pdf [file cm3c03110_si_001.pdf]

Supporting Information: **Multinary Tetrahedrite ($Cu_{12-x-y}M_xN_ySb_4S_{13}$) Nanoparticles: Tailoring Thermal and Optical Properties with Copper-site Dopants**

Jacob E. Daniel,† Christian M. Jesby,‡ Katherine E. Plass,‡ and Mary E. Anderson†*

† Chemistry Department, Furman University, Greenville, SC 29613 US
‡ Chemistry Department, Franklin & Marshall College, Lancaster, PA 17604 US
* Corresponding Author: maryelizabeth.anderson@furman.edu

Included:

Table S1: Rietveld refinement calculations for the lattice parameter *a* and grain size of undoped, single-doped, and co-doped tetrahedrite nanoparticles. (Page 2)

Figures S1-S6: Rietveld refinement plots for undoped single-doped tetrahedrite nanoparticles (Page 3-8)

Size Distribution Analysis: Process description for analyzing size and shape of tetrahedrite nanoparticles. (Page 9)

Figure S7: TEM images and size distribution histogram for $Cu_{11}Fe_{0.5}Mn_{0.5}Sb_4S_{13}$ co-doped sample. (Page 10)

Figure S8: Raw optical absorbance spectra for undoped and single-doped tetrahedrite nanoparticles. (Page 11)

Table S2: Summary of band gap values derived from linear fits of $(A h\nu)^2$ for undoped and single-doped tetrahedrite nanoparticle samples. (Page 12)

Figure S9: Tauc plots of $(A h\nu)^2$ for co-doped tetrahedrite combinations. (Page 13)

Table S3: Summary of band gap values derived from linear fits of $(A h\nu)^2$ for co-doped tetrahedrite nanoparticle samples. (Page 14)

Figure S10: Thermal gravimetric data of co-doped tetrahedrite nanoparticles plotted with thermal gravimetric data of relevant single-doped nanomaterials (Page 15)

Figure S11: Normalized absorbance spectra of co-doped tetrahedrite nanoparticles plotted alongside the normalize absorbance spectra of relevant single-doped nanomaterials. (Page 16)

Rietveld refinements for undoped, single-doped, and co-doped tetrahedrite[a]

Abbreviation	Target	a (Å)	Grain Size (Å)	Chi^{2} [b]
Undoped	$Cu_{12}Sb_4S_{13}$	10.3403 (11)	166.4 (19)	1.0074
Zn_1	$Cu_{11}ZnSb_4S_{13}$	10.3611 (17)	166.0 (30)	1.5030
Fe_1	$Cu_{11}FeSb_4S_{13}$	10.3511 (11)	188.3 (18)	1.6869
Ni_1	$Cu_{11}NiSb_4S_{13}$	10.3249 (9)	154.4 (20)	1.0881
Mn_1	$Cu_{11}MnSb_4S_{13}$	10.3960 (20)	151.6 (12)	1.1069
Co_1	$Cu_{11}CoSb_4S_{13}$	10.3408 (11)	177.9 (18)	1.0436
$Zn_{0.5}Fe_{0.5}$	$Cu_{11}Zn_{0.5}Fe_{0.5}Sb_4S_{13}$	10.3606 (10)	187.0 (30)	1.0327
$Zn_{0.5}Ni_{0.5}$	$Cu_{11}Zn_{0.5}Ni_{0.5}Sb_4S_{13}$	10.3378 (9)	198.0 (20)	1.0463
$Zn_{0.5}Mn_{0.5}$	$Cu_{11}Zn_{0.5}Mn_{0.5}Sb_4S_{13}$	10.3631 (9)	196.0 (30)	1.0417
$Zn_{0.5}Co_{0.5}$	$Cu_{11}Zn_{0.5}Co_{0.5}Sb_4S_{13}$	10.3563 (11)	145.3 (20)	1.0946
$Fe_{0.5}Ni_{0.5}$	$Cu_{11}Fe_{0.5}Ni_{0.5}Sb_4S_{13}$	10.3415 (9)	207.0 (30)	1.0497
$Fe_{0.5}Mn_{0.5}$	$Cu_{11}Fe_{0.5}Mn_{0.5}Sb_4S_{13}$	10.3709 (10)	220.0 (30)	1.0253
$Fe_{0.5}Co_{0.5}$	$Cu_{11}Fe_{0.5}Co_{0.5}Sb_4S_{13}$	10.3469 (13)	223.0 (20)	1.5488
$Ni_{0.5}Mn_{0.5}$	$Cu_{11}Ni_{0.5}Mn_{0.5}Sb_4S_{13}$	10.3534 (10)	243.0 (11)	1.1405
$Ni_{0.5}Co_{0.5}$	$Cu_{11}Ni_{0.5}Co_{0.5}Sb_4S_{13}$	10.3359 (11)	215.0 (30)	1.5134
$Mn_{0.5}Co_{0.5}$	$Cu_{11}Mn_{0.5}Co_{0.5}Sb_4S_{13}$	10.3536 (17)	183.0 (30)	1.1058

[a]Rietveld refinement performed using PDXL2 software
[b]Chi^{2} is a goodness-of-fit measurement.

Table S1: Calculated lattice parameter (a), grain sizes, and chi-squared values along with associated error in parentheses for all tetrahedrite samples as determined by Rietveld refinement. Reference pattern for $Cu_{12}Sb_4S_{13}$ (PDF# 01-071-0270) was utilized for Rietveld refinement performed using PDXL2 software.[1] Throughout the manuscript, tetrahedrite samples are referred to by abbreviations listed in the first column of this table.

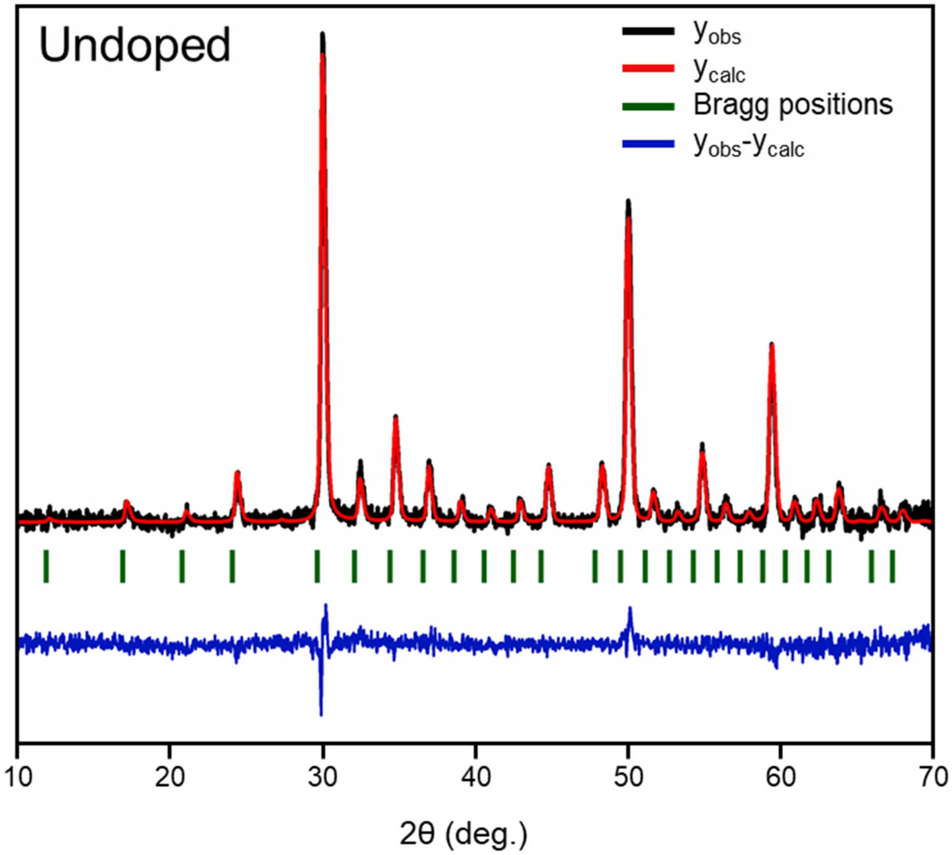

Figure S1: Rietveld refinement plot of the undoped tetrahedrite nanoparticles. Bragg positions were obtained from PDF Card #01-074-0270.[1]

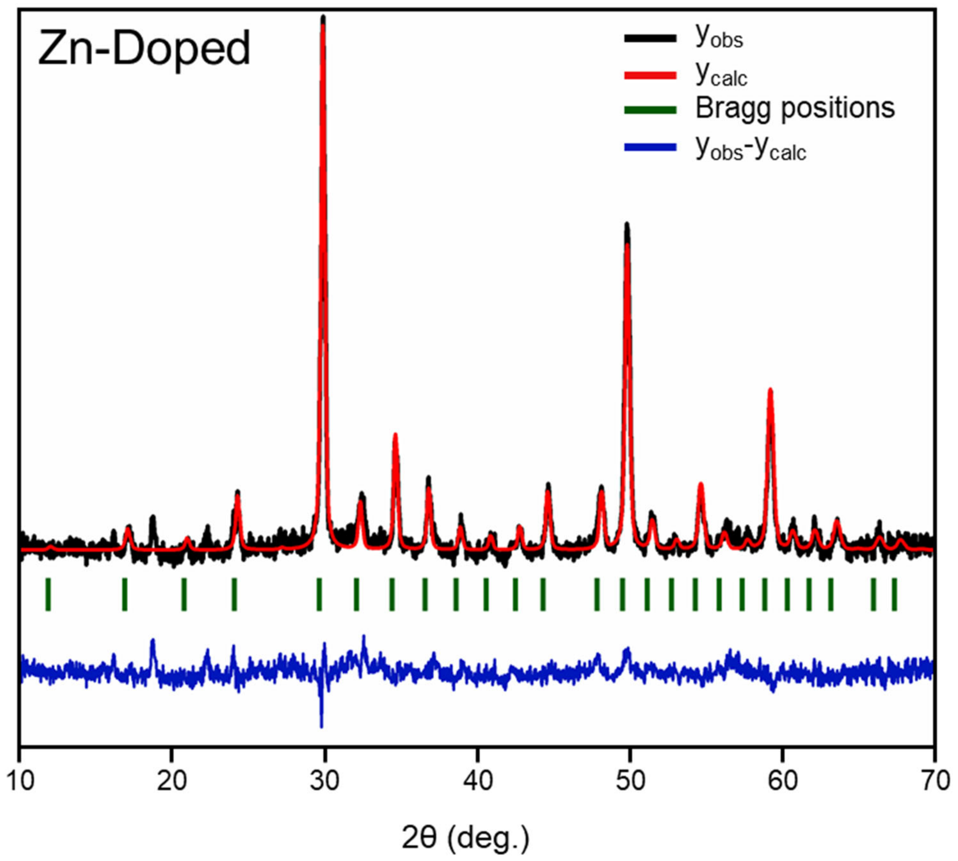

Figure S2: Rietveld refinement plot for the Zn-doped tetrahedrite nanoparticles. Bragg positions were obtained from PDF Card #01-074-0270.[1]

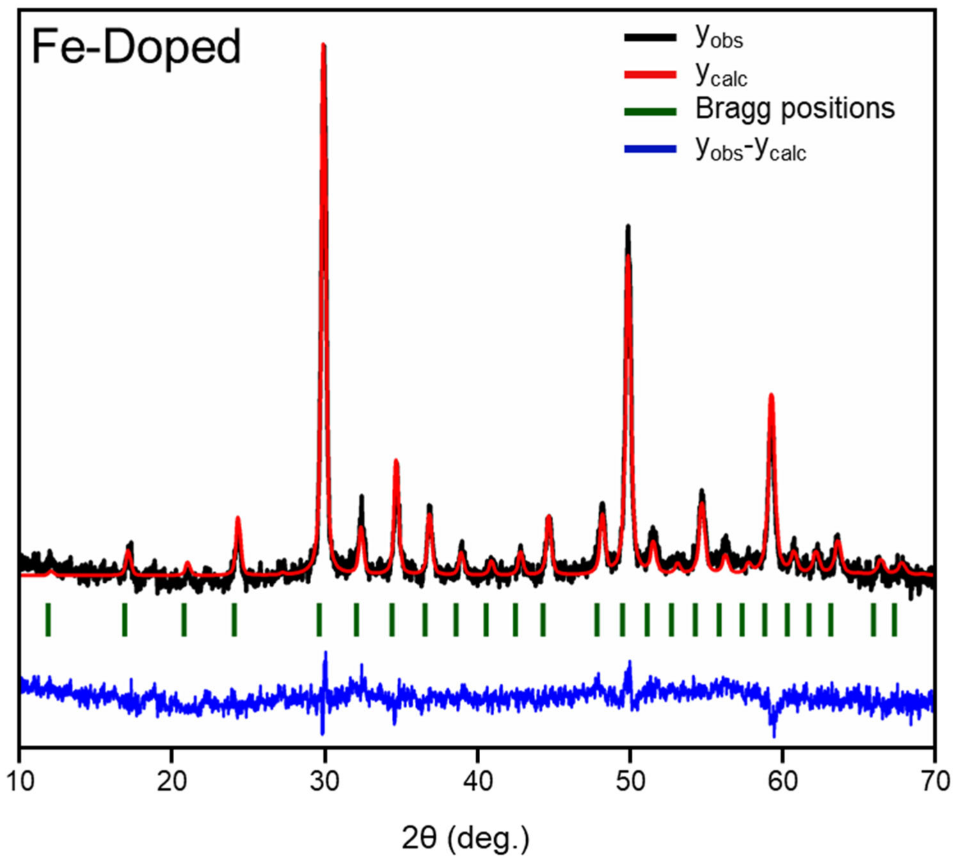

Figure S3: Rietveld refinement plot for the Fe-doped tetrahedrite nanoparticles. Bragg positions were obtained from PDF Card #01-074-0270.[1]

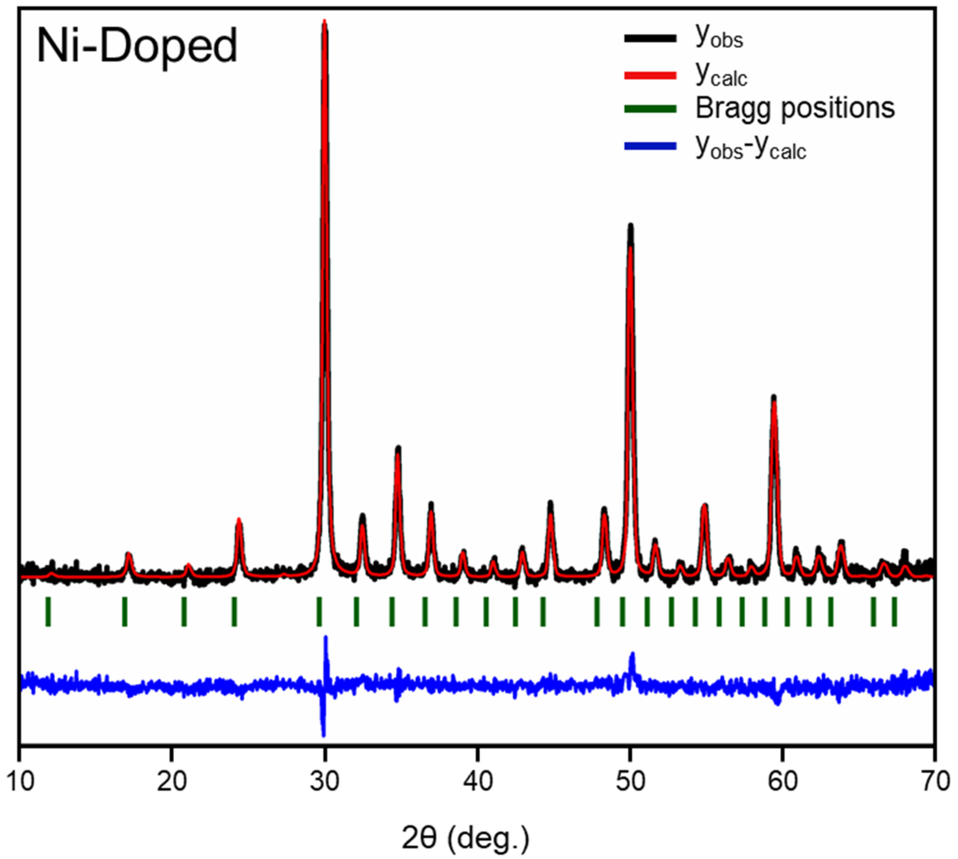

Figure S4: Rietveld refinement plot for the Ni-doped tetrahedrite nanoparticles. Bragg positions were obtained from PDF Card #01-074-0270.[1]

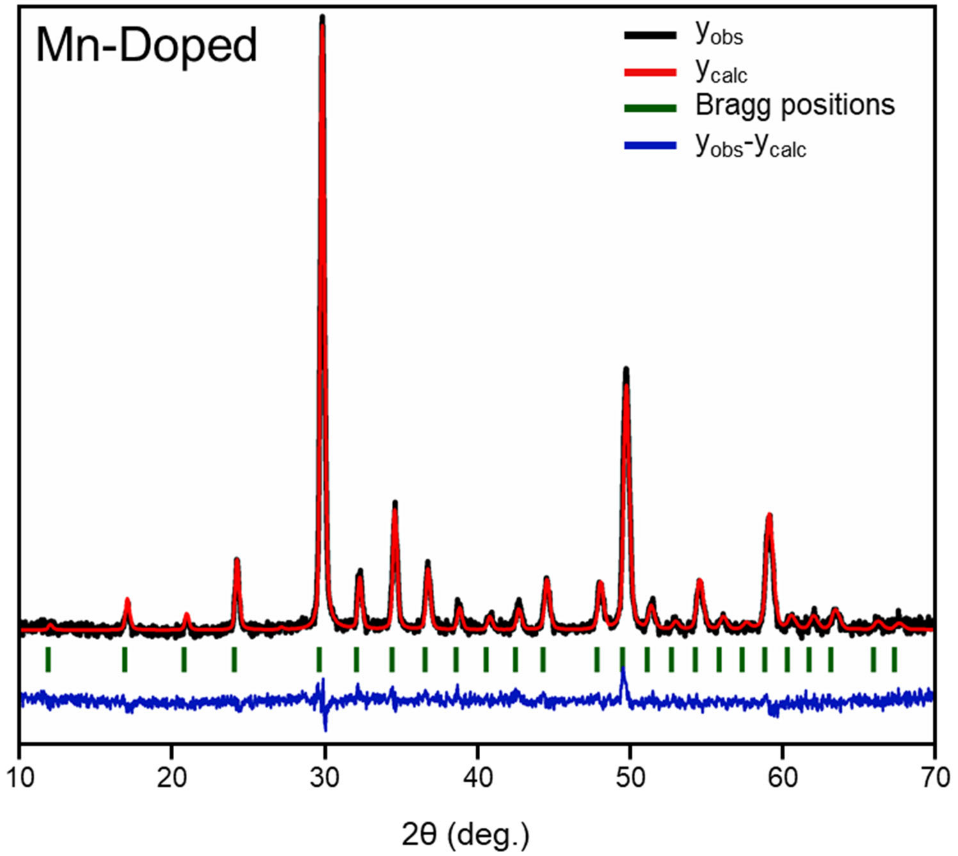

Figure S5: Rietveld refinement plot for the Mn-doped tetrahedrite nanoparticles. Bragg positions were obtained from PDF Card #01-074-0270.[1]

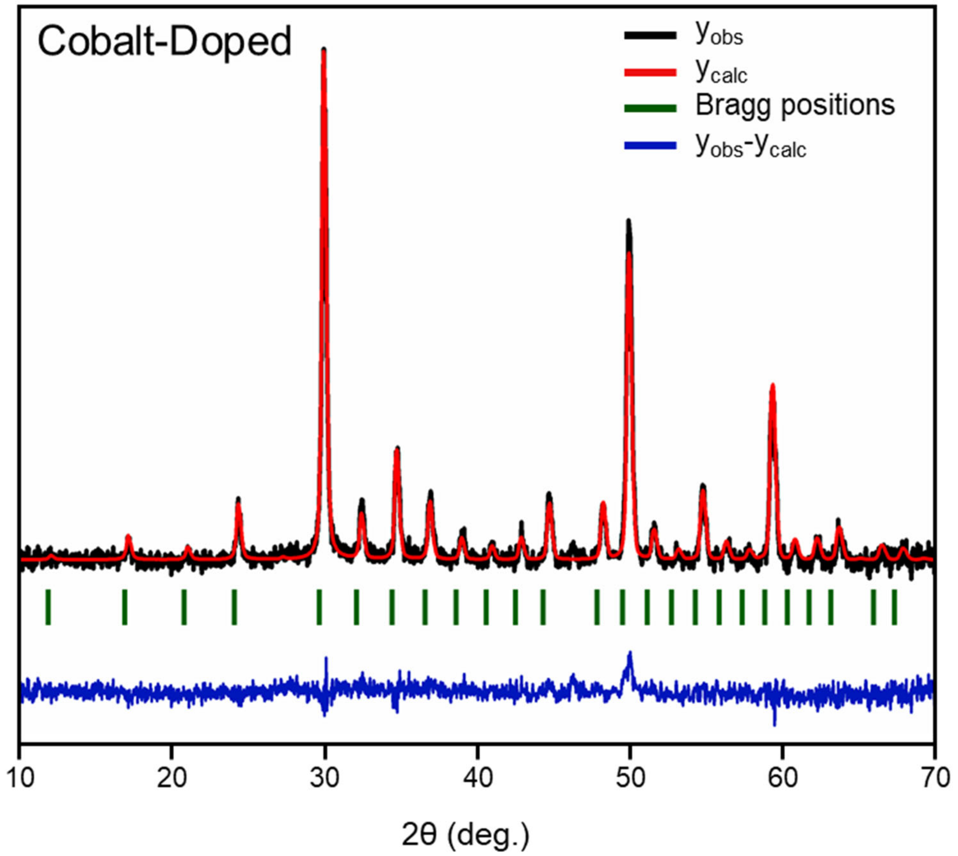

Figure S6: Rietveld refinement plot for the cobalt-doped tetrahedrite nanoparticles. Bragg positions were obtained from PDF Card #01-074-0270.[1]

Size Analysis Procedure:

Transmission electron microscopy **(Fig. S7a-b)** shows $Fe_{0.5}Mn_{0.5}$ co-doped tetrahedrite nanoparticles are not solely spherical in shape, but instead vary from spherical to ovoid. Clusters of particles appear in TEM images, but individual particles can be distinguished for image analysis. The $Fe_{0.5}Mn_{0.5}$ co-doped tetrahedrite nanoparticles exhibit lengths of 110 ± 20 nm. The histogram shown in **Fig. S7c** provides the full range of lengths for all nanoparticles sampled. Nanoparticle size measurements are taken using the Fiji software. After setting the image scale, lines were drawn connecting the longest distance of two edges as shown in **Fig. S7d**. The distances of those lines were saved and analyzed using Excel.

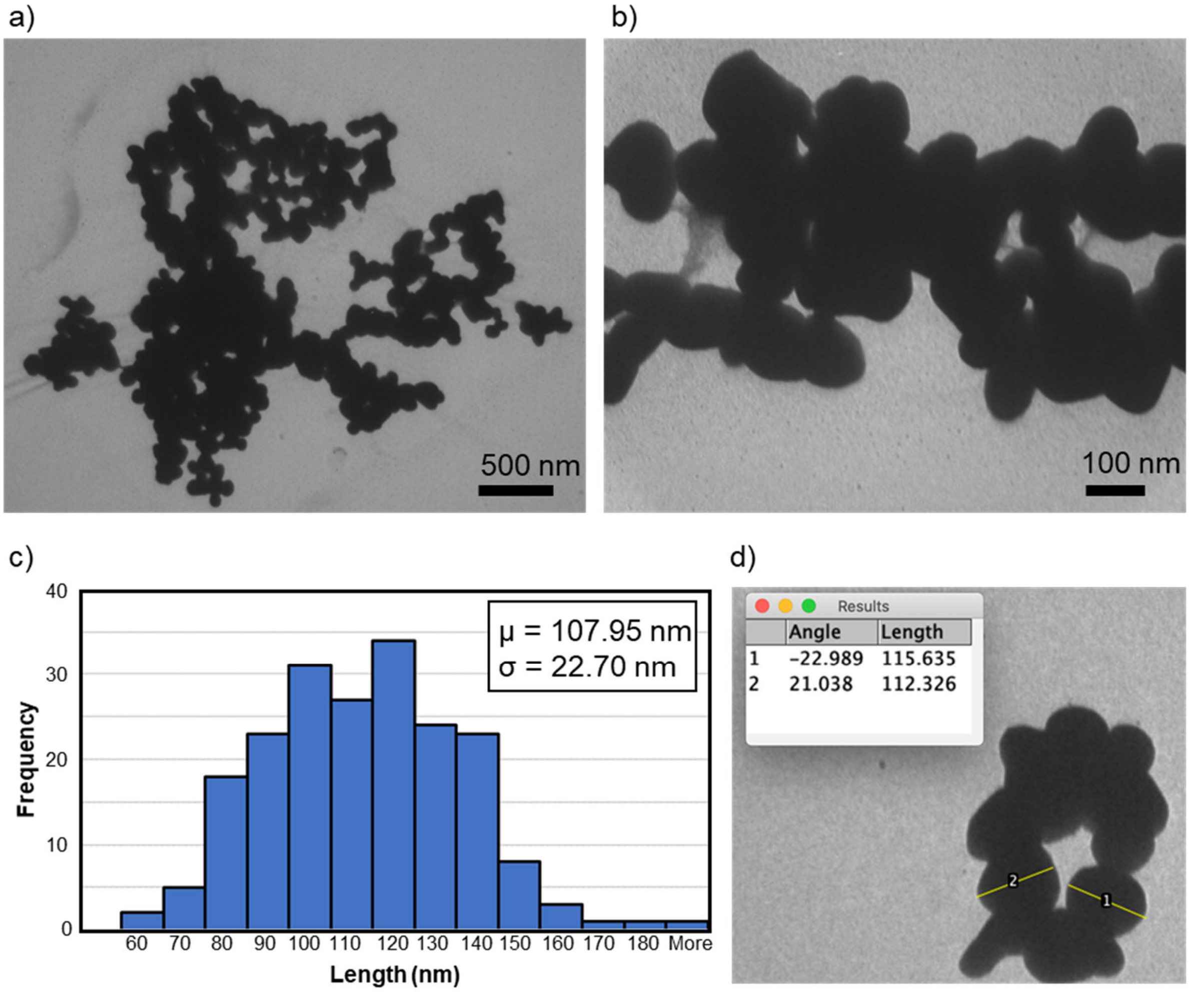

Figure S7: (a-b) Representative TEM images of the $Cu_{11}Fe_{0.5}Mn_{0.5}Sb_4S_{13}$ co-doped tetrahedrite nanoparticles alongside (c) size distribution histogram for the $Cu_{11}Fe_{0.5}Mn_{0.5}Sb_4S_{13}$ sample with average nanoparticle size and the standard deviation inset. (d) An example of how nanoparticle size was measured using Fiji software.

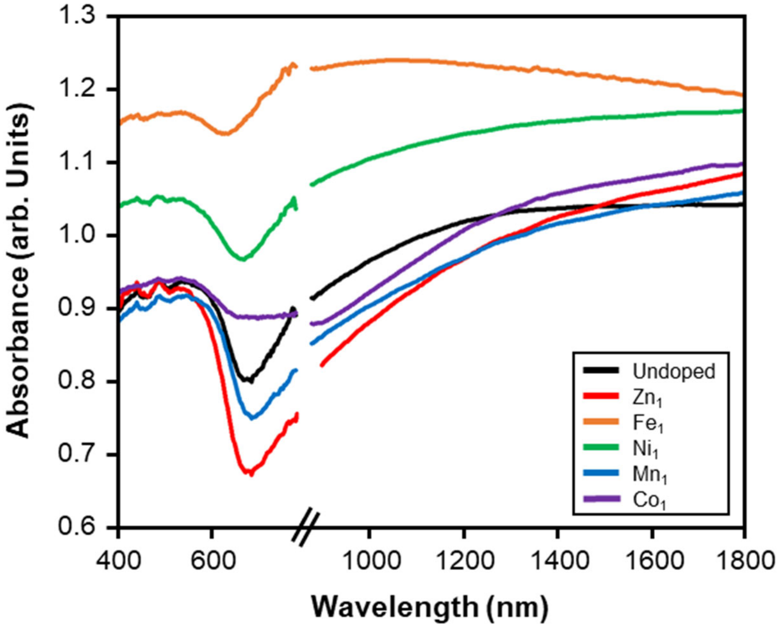

Figure S8: Raw optical absorbance spectra of undoped ($Cu_{12}Sb_4S_{13}$) and single-doped ($Cu_{11}M_1Sb_4S_{13}$, *M*=Zn, Fe, Ni, Mn, or Co) nanoparticles. The x-axis breaks from 785 nm to 875 nm due to a detector changeover.

Summary of $(A\text{hv})^2$ Tauc plots for undoped and single doped nanoparticles

Sample	Direct Band Gap (eV)	Linear Fit	R^2
Undoped	1.90	y = 2.764x – 5.261	0.997
Zn_1	1.92	y = 2.496x – 4.792	0.999
Fe_1	2.04	y = 3.634x – 7.430	0.998
Ni_1	1.96	y = 2.554x – 5.001	0.999
Mn_1	1.88	y = 2.573x – 4.825	0.991
Co_1	1.95	y = 2.785x – 5.425	0.998

Table S2: Direct band gap values are presented alongside linear fit information for the $(A\text{hv})^2$ Tauc plots analyzing the optical spectra of undoped ($Cu_{12}Sb_4S_{13}$) and single-doped ($Cu_{11}M_1Sb_4S_{13}$, *M*=Zn, Fe, Ni, Mn, or Co) tetrahedrite nanoparticles. Normalized absorbance spectra and Tauc plots with linear fit shown in **Fig. 6**.

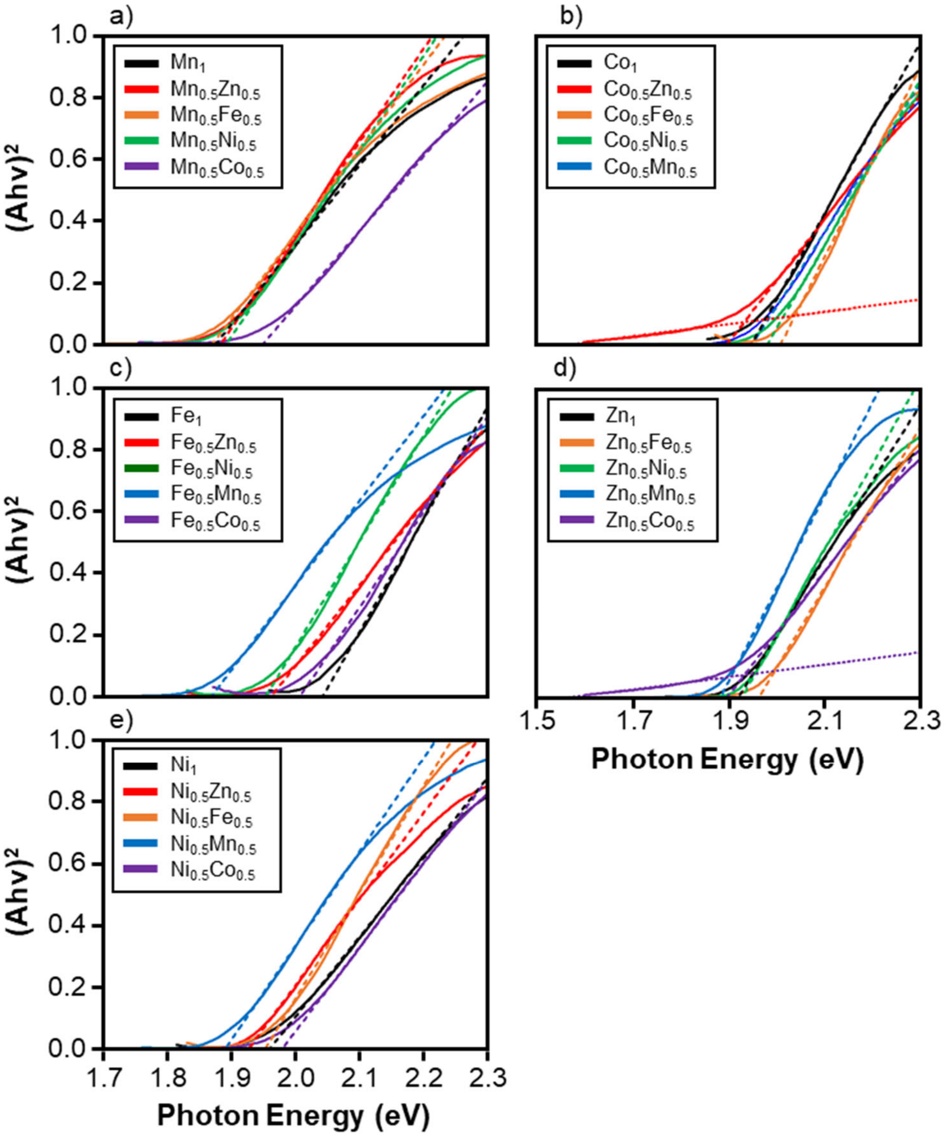

Figure S9: Tauc plots of $(Ah\nu)^2$ for the (a) Mn, (b) Co, (c) Fe, (d) Zn, and (e) Ni co-doped tetrahedrite ($Cu_{11}M_{0.5}N_{0.5}Sb_4S_{13}$; *M, N* = Zn, Fe, Ni, Mn, Co) combinations alongside the relevant single-doped sample. The x-intercept of the linear fits for the Tauc plots in (a-e) yields the magnitude of the optical band gap, found in **Table S3** and **Fig. 7c**.

Summary of $(A h\nu)^2$ Tauc plots for co-doped nanoparticles

Sample	Direct Band Gap (eV)	Linear Fit	R^2
$Zn_{0.5}Fe_{0.5}$	1.96	y = 2.601x – 5.107	0.995
$Zn_{0.5}Ni_{0.5}$	1.93	y = 2.769x – 5.340	0.999
$Zn_{0.5}Mn_{0.5}$	1.88	y = 3.006x – 5.660	0.996
$Zn_{0.5}Co_{0.5}$	1.58<br>1.90	y = 2.005x – 3.805<br>y = 0.199x – 0.314	0.999<br>0.998
$Fe_{0.5}Ni_{0.5}$	1.95	y = 3.412x – 6.669	0.995
$Fe_{0.5}Mn_{0.5}$	1.86	y = 2.755x – 5.114	0.999
$Fe_{0.5}Co_{0.5}$	2.01	y = 3.091x – 6.209	0.995
$Ni_{0.5}Mn_{0.5}$	1.89	y = 3.016x – 5.705	0.999
$Ni_{0.5}Co_{0.5}$	1.98	y = 2.694x – 5.337	0.999
$Mn_{0.5}Co_{0.5}$	1.98	y = 4.029x – 7.996	0.999

Table S3: Direct band gaps values are presented alongside linear fit information for the $(A h\nu)^2$ Tauc plots **(Fig. S9)** analyzing the optical spectra of the co-doped ($Cu_{11}M_{0.5}N_{0.5}Sb_4S_{13}$, *M/N*=Zn, Fe, Ni, Mn, or Co) tetrahedrite nanoparticles. There are two linear regions in the Tauc plot of the $Zn_{0.5}Co_{0.5}$ combination which resulted in two band gaps.

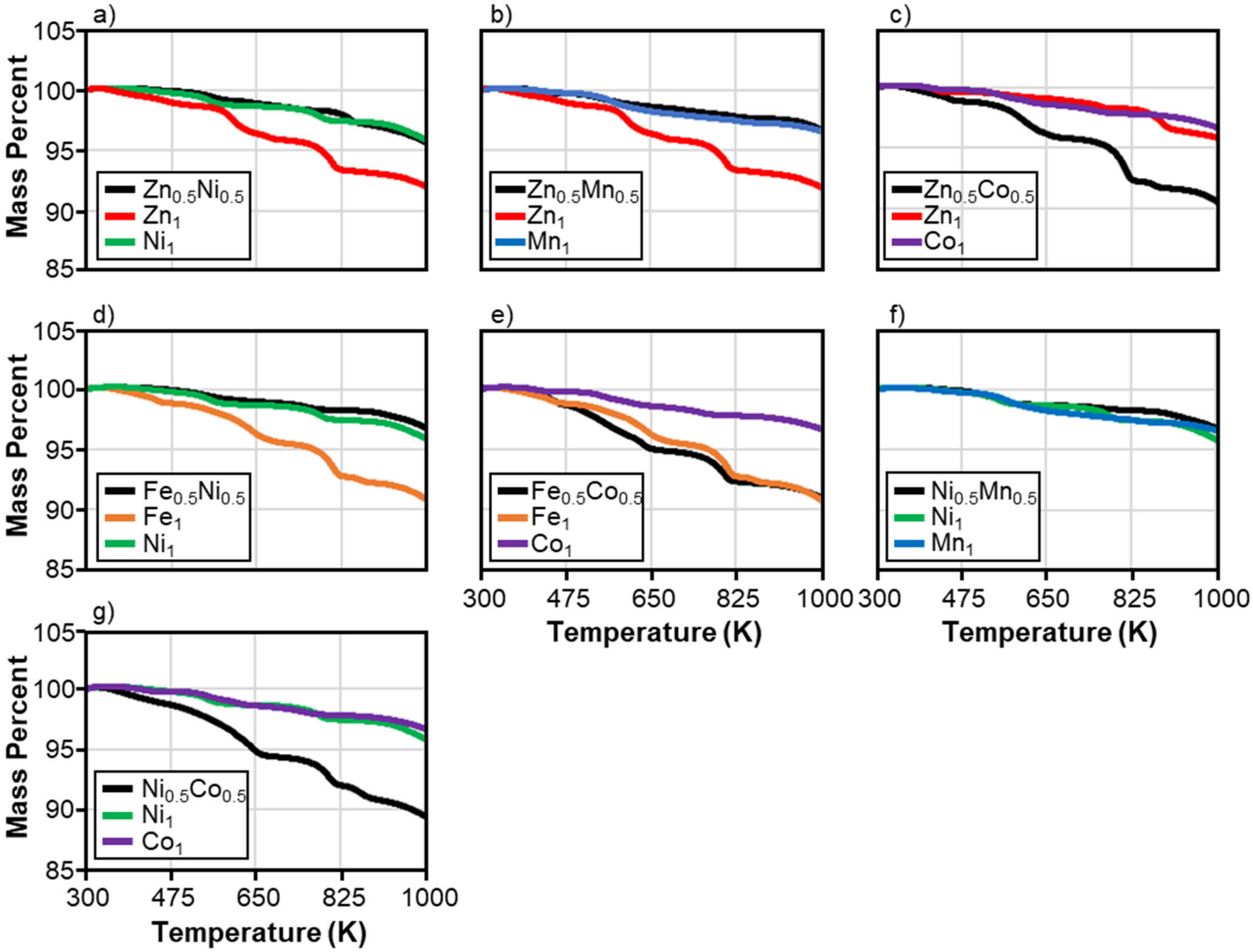

Figure S10: Thermal gravimetric curves for the (a) $Zn_{0.5}Ni_{0.5}$, (b) $Zn_{0.5}Mn_{0.5}$, (c) $Zn_{0.5}Co_{0.5}$, (d) $Fe_{0.5}Ni_{0.5}$, (e) $Fe_{0.5}Co_{0.5}$, (f) $Ni_{0.5}Mn_{0.5}$, and (g) $Ni_{0.5}Co_{0.5}$ co-doped nanoparticles alongside the thermal gravimetric curves for the two relevant single-doped nanoparticles. Data for the $Zn_{0.5}Fe_{0.5}$, $Fe_{0.5}Mn_{0.5}$, and $Mn_{0.5}Co_{0.5}$ combinations are provided in **Fig. 8**.

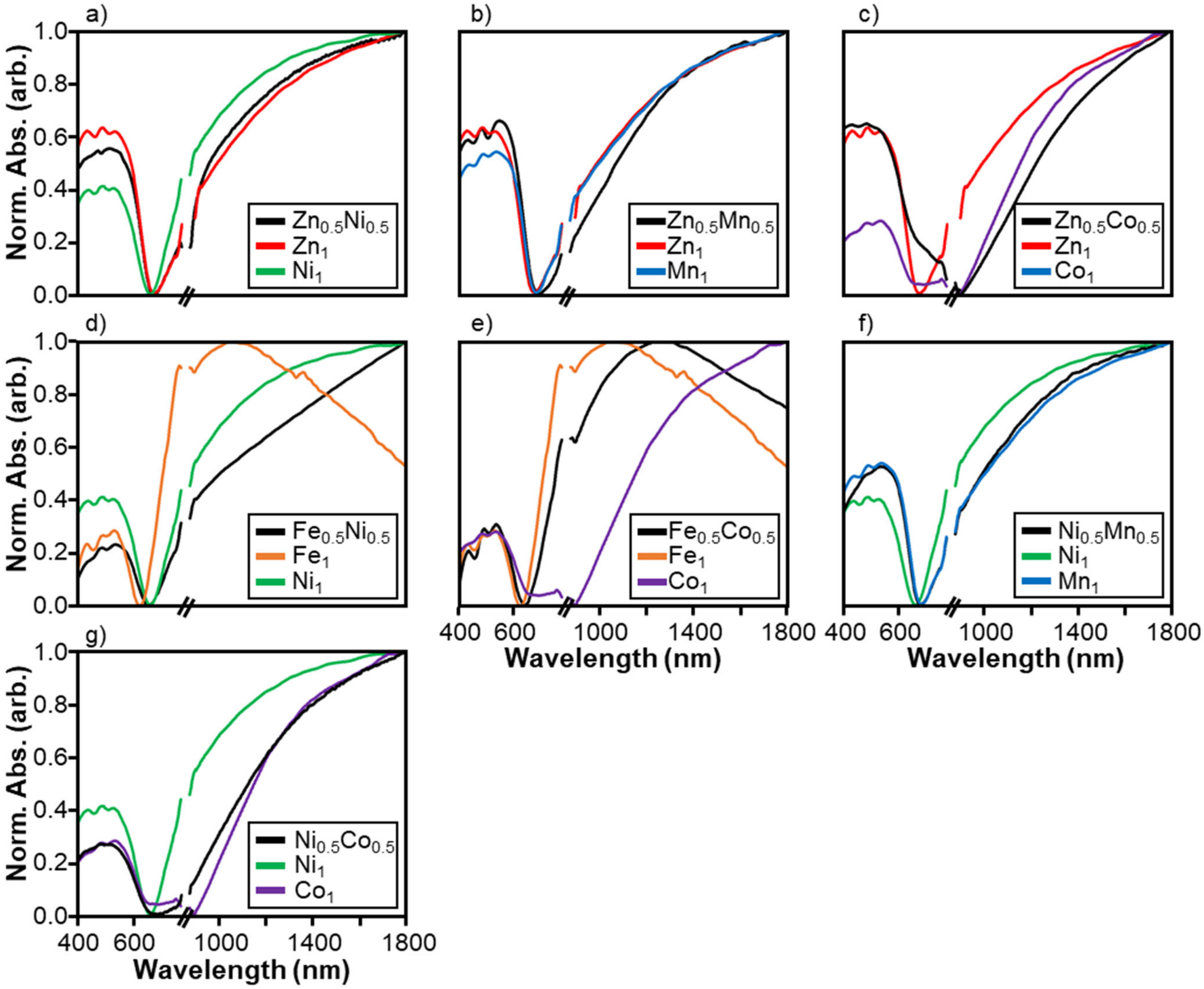

Figure S11: Optical spectra for the (a) $Zn_{0.5}Ni_{0.5}$, (b) $Zn_{0.5}Mn_{0.5}$, (c) $Zn_{0.5}Co_{0.5}$, (d) $Fe_{0.5}Ni_{0.5}$, (e) $Fe_{0.5}Co_{0.5}$, (f) $Ni_{0.5}Mn_{0.5}$, and (g) $Ni_{0.5}Co_{0.5}$ co-doped nanoparticles alongside the optical spectra of the two relevant single-doped nanoparticles. Data for the $Zn_{0.5}Fe_{0.5}$, $Fe_{0.5}Mn_{0.5}$, and $Mn_{0.5}Co_{0.5}$ combinations are provided in **Fig. 8**.

Reference

1. Wuensch, B. J. *Zeitschrift fuer Kristallographie, Kristallgeometrie, Kristallphysik, Kristallchemie*. **1964**, *119*, 437-453.
